# Caspase-mediated AURKA cleavage at Asp^132^ is essential for paclitaxel to elicit cell apoptosis

**DOI:** 10.7150/thno.97842

**Published:** 2024-06-17

**Authors:** Xiaoting Chen, Shujuan Du, Yulin Zhang, Ke Peng, Lina Liu, Ting Wang, Hao Zhang, Shen Cai, Caixia Zhu, Youhai Li, Wen Tuo, Yuyan Wang, Fang Wei, Qiliang Cai

**Affiliations:** 1 MOE/NHC/CAMS Key Laboratory of Medical Molecular Virology, Shanghai Institute of Infections Disease and Biosecurity, Shanghai Frontiers Science Center of Pathogenic Microorganism and Infection, School of Basic Medical Science, Shanghai Medical College, Fudan University, Shanghai 200032, P. R. China.; 2 ShengYushou Center of Cell Biology and Immunology, School of Life Sciences and Biotechnology, Shanghai Jiao Tong University, Shanghai 200240, P. R. China.; 3 Baoji Central Hospital, Baoji, People's Republic of China.; 4 Expert Workstation, Baoji Central Hospital, Baoji, People's Republic of China.

**Keywords:** AURKA, Proteolytic Cleavage, Caspase, Apoptosis, Chemotherapeutic Drug

## Abstract

**Background:** Aurora kinase A (AURKA) is a potent oncogene that is often aberrantly expressed during tumorigenesis, and is associated with chemo-resistance in various malignancies. However, the role of AURKA in chemo-resistance remains largely elusive.

**Methods:** The cleavage of AURKA upon viral infection or apoptosis stimuli was assesed by immunoblotting assays in several cancer cells or caspase deficient cell line models. The effect of AURKA cleavage at Asp^132^ on mitosis was explored by live cell imaging and immunofluorescence staining experiments. The role of Asp132-cleavage of AURKA induced by the chemotherapy drug paclitaxel was investigated using TUNEL, immunohistochemistry assay in mouse tumor xenograft model and patient tissues.

**Results:** The proteolytic cleavage of AURKA at Asp^132^ commonly occurs in several cancer cell types, regardless of viral infection or apoptosis stimuli. Mechanistically, caspase 3/7/8 cleave AURKA at Asp^132^, and the Asp^132^-cleaved forms of AURKA promote cell apoptosis by disrupting centrosome formation and bipolar spindle assembly in metaphase during mitosis. The AURKA^D132A^ mutation blocks the expression of cleaved caspase 3 and EGR1, which leads to reduced therapeutic effects of paclitaxel on colony formation and malignant growth of tumor cells *in vitro* and *in vivo* using a murine xenograft model and cancer patients.

**Conclusions:** This study reveals that caspase-mediated AURKA^D132^ proteolysis is essential for paclitaxel to elicit cell apoptosis and indicates that AURKA^D132^ is a potential key target for chemotherapy.

## Introduction

Cancer is a major threat to human health and is one of the leading causes of deaths worldwide. Intrinsic or acquired resistance to apoptosis is one of the hallmarks of human cancer 1. Most currently available cancer therapies, including chemotherapy, radiotherapy, and immunotherapy, elicit therapeutic effects by inducing apoptosis of cancer cells 2. Chemotherapy is commonly used to treat various cancers, however, drug resistance caused by defective apoptosis hinders the efficacy of chemotherapy 2. Understanding the mechanisms underlying tumor cell death in response to chemotherapeutic drugs is essential for improving the efficacy of chemotherapy.

Aurora kinase A (AURKA) is a potent oncogene and an attractive target for cancer therapeutics. AURKA belongs to the serine and threonine kinase family 3. In mammals, the AURK kinase family includes AURKA, AURKB, and AURKC 4, 5. AURKA and AURKB play important roles in the regulation of mitotic cell division, and AURKC has a unique physiological role in meiosis. The human AURKA gene is located on chromosome 20q13. The gene has an open reading frame of 1209 bp, which encodes a protein of 403 amino acids with a molecular weight of 46 kDa 6. AURKA plays key roles in mitosis by regulating centrosome maturation and separation 7, bipolar spindle assembly 8, mitotic entry, and cytokinesis 9. In addition, AURKA regulates non-mitotic functions, including flagellar disassembly 10, neurite extension, calcium regulation, mitochondrial dynamics, and energy production 11. AURKA is aberrantly expressed in a wide range of human cancers, including breast cancer 12, ovarian cancer 13, cervical cancer 14, and hepatocellular carcinoma 15, and the aberrant expression of AURKA correlates with poor prognosis. AURKA overexpression or aberrant activation may lead to chromosome instability during mitosis, resulting in aberrant cell cycle regulation, tumorigenesis, and drug resistance 16. AURKA overexpression induces chemoresistance in gastric cancer 17, ovarian 18, and breast cancer patients 19 by upregulating the expression of survivin or activating Akt in a p53-dependent manner.

AURKA plays an important role in regulating tumor cell death and is promising therapeutic target 20, 21. For example, the knockdown of AURKA sensitizes human colorectal cancer to radiation 16. Inhibition of AURKA triggers autophagic cell death and enhances radio-sensitivity in non-small cell lung cancer 22. Silencing AURKA downregulates the SRC-mediated ERK and mTOR pathways to increase breast cancer cell sensitivity to paclitaxel (Taxol) 23. AURKA inhibition and Taxol treatment synergistically increase apoptosis in head and neck squamous cell carcinoma cells 24. Additionally, chemotherapy drugs such as JQ1, camptothecin, and 5-fluorouracil directly or indirectly reduce AURKA expression in the liver, cervical, and breast cancer cell lines, respectively 25. These findings all focused on reducing the expression of AURKA as a promising target for cancer therapy. Tumor growth and metastasis are complex, and most tumors do not rely on a single signaling pathway to sustain progression. Hence, understanding the mechanisms of AURKA actions is essential for improving the efficacy of anti-tumor therapy and overcoming drug resistance.

Viral infection can induce the extrinsic apoptosis pathway, which plays an important role in the host antiviral response and viral oncogenesis. In addition, host cells eliminate virus-infected cells through apoptosis to abort viral infections 26. However, some viruses release and spread progeny viruses via apoptosis. Kaposi's sarcoma-associated herpesvirus (KSHV), also known as human herpesvirus 8, was first identified by Chang and Moore in Kaposi's sarcoma (KS) tissues from AIDS patients. KSHV is associated with several human malignancies, including KS 27 and primary effusion lymphoma 28. The KSHV-encoded latency-associated Nuclear Antigen (LANA) is expressed during KSHV latency and plays a key role in viral DNA replication, ensuring the proper segregation of replicated genomes into daughter cells 29. We previous demonstrated that the LANA protein upregulates AURKA mRNA levels by targeting the Sp1 cis-element within the promoter, leading to p53 phosphorylation and ubiquitylation 30. We also discovered that LANA induces AURKB cleavage at Asp^76^ in a serine protease-dependent manner to promote cell segregation and tumorigenesis. However, AURKA cleavage and the related molecular mechanisms remain elusive.

In this study, we demonstrate that overexpression of LANA protein or treatment with apoptotic stimuli leads to AURKA cleavage at Asp^132^. This cleavage is selectively inhibited by caspase 3/7/8 inhibition but not inhibitors of caspase 1 or other serine proteases. Apoptotic stimuli lead to Asp^132^-cleavage of AURKA in various human cancer cell lines, regardless of viral infection. Caspase 3/7/8, but not caspase 6, is required for Asp^132^-cleavage of AURKA, and the Asp^132^-cleavage products significantly disrupt centrosome formation and spindle assembly in metaphase during mitosis, leading to cell apoptosis. Mutant of AURKA^D132A^ blocks the expression of cleaved caspase 3 and EGR1, and prevents Taxol-induced apoptosis of cancer cells *in vitro*. In a murine xenograft model, the efficacy of chemotherapy is decreased by the AURKA^D132A^ mutation. Overall, our findings indicate that Asp^132^-cleavage of AURKA is a potential key biomarker for the efficacy of chemotherapeutic drugs.

## Materials and Methods

**Human Subjects —** Breast tissue sections from female breast cancer patients (age 30 to 73) treated with paclitaxel chemotherapy were all collected from Baoji Central Hospital (Baoji City, Shanxi Province, China). The Miller-Payne (MP) grading system (from G1 to G5) was used to evaluate the sensitivity of neoadjuvant chemotherapy (NAC) in the breast cancer patients, according to the proportion of residual cells of the breast primary tumor after and before NAC treatment in the biopsy specimen. Total eighteen tissue samples from different patients with 4-weeks treatment were collected with two groups: Grade 2 (insensitive to Taxol-based chemotherapy with less than 30% reduction of cancer cells after NAC, n=9) and Grade 5 (sensitive to Taxol-based chemotherapy without viable cancer cells observed within resected tumor after NAC, n=9). More detail descriptions of patient characteristics and chemotherapy regimens are provided in Supplementary Table S1. Usage of redundant cancer sample for research purpose was approved by the Hospital Medical Ethics Committee (No. BZYL2023-46, 2023-8-25). The IRB approved protocol in which Declaration of Helsinki protocols were followed and each donor gave written, informed consent. Sample size was based on feasibility and availability of human excess tissue collections.

**Cell Lines —** HEK293 (ATCC, CRL-1537), HEK293T (CCTCC, GDC187), human cervical cancer cells HeLa (ATCC, CCL-2), human breast cancer cells MDA-MB-231(ATCC, HBT-26), human hepatocellular carcinoma cells SMMC-7721 (kindly provided by Jian Wu from Fudan university) 31 and HLE (kindly provided by Jian Wu from Fudan university) were cultured in DMEM with 6% FBS and penicillin-streptomycin. KSHV negative iSLK (1 µg/ml Puromycin, 250 µg/ml G418) and KSHV positive iSLK-Bac16 (1.2 mg/ml Hygromycin, 1 µg/ml Puromycin, 250 µg/ml G418, kindly provided by Shou-Jiang Gao from University of Pittsburgh) cells were maintained in DMEM supplemented with 6% FBS 32. KSHV negative MM and KSHV positive KMM cells (kindly provided by Shou-Jiang Gao from University of Pittsburgh) were maintained in DMEM contained 6% FBS 33. EBV-negative DG75 (ATCC, CRL-2625), EBV-positive B95.8 (ATCC, CRL-1612), KSHV-positive BC3 (ATCC, CRL-2277) and BCBL1, KSHV-negative BJAB (kindly provided by Erle Robertson from University of Pennsylvania) were maintained in RPMI1640 medium supplemented with 8% FBS and penicillin-streptomycin as described previously 34. All cell lines were incubated at 37˚C in a humidified environmental incubator with 5% CO2.

**Animal Ethics Statement and Husbandry —**All animal studies were conducted in accordance with the China Guide for the Care and Use of Laboratory Animals. All experiments were approved and supervised by the Institutional Animal Care and Use committee at Fudan University under Protocol ID 20190221-026. Five-week-old female BALB/c nude mice were purchased from Beijing Vital River Laboratory Animal Technology Co., Ltd (Beijing, China). Mice were housed in microisolator cages with no more than five mice per cage at certified specific-pathogen-free or germ-free vivaria. Animals were provided with autoclaved water and food *ad libitum.*

**Antibodies and Reagents —** Mouse monoclonal antibodies against GFP (F56-6A1, sc-53882), PARP1 (F-2, sc-8007), α-tubulin (1E4C11, Proteintech), FLAG (M2, Sigma-Aldrich), GAPDH (G8140-01, US Biological), caspase-6 (1H7F9, Proteintech), caspase-8 (2B9H8, Proteintech). Rabbit monoclonal antibodies against AURKA-C (EP1008Y, ab52973, abcam.1:5,000). Rabbit polyclonal antibodies against caspase-3 (H-277, sc-7148), caspase-7 (27155-1-AP, Proteintech). The monoclonal antibodies against myc (9E10) were individually prepared from hybridoma cultures. Secondary antibodies, IRDye 800cw Goat Anti-Mouse IgG and IRDye 800cw Goat Anti-Rabbit IgG were used at 1:10,000 dilution. Protease inhibitors PMSF, aprotinin, leupeptin, and pepstatin A were purchased from Amresco. Caspase inhibitors Z-VAD-FMK (CAS 187389-52-2), and Z-DEVD-FMK (CAS 210344-95-9) were purchased from Target Mol, and Ac-YVAD-CHO was purchased from Santa Cruz. Apoptosis-inducing drugs paclitaxel (HY-B0015) and staurosporine (HY-15141) were purchased from MCE.

**DNA Constructs —** Plasmid expression full-length AURKA was generated by PCR amplicon (with pcDNA4-myc-AURKA as template) and inserted into pA3M vector through *Bam*HI and *Xho*I sites, pEGFP-C1 vector through *Xho*I and *Bam*HI sites, respectively to construct AURKA-myc and GFP-AURKA plasmids. AURKA site mutants D132A, K141A, D294A, and D350A were individually constructed by PCR-directed site mutation with AURKA-myc and GFP-AURKA as templates. The plasmid expressing AURKA^1-132^ was individually obtained by PCR-directed site mutation with GFP-AURKA, and the truncation mutant AURKA^133-end^ was constructed by PCR amplicon (pA3M-AURKA as template) inserted into pEGFP-C1 vector with restriction enzymes *Kpn*I and *Bam*HI sites, respectively. The expression plasmids pLVX-YFP-AURKA^WT^, pLVX-YFP-AURKA^1-132^, pLVX-YFP-AURKA^133-end^ and pLVX-YFP-AURKA^D132A^ were generated by PCR amplicon inserted into pLVX-YFP vector (modified from pLVX-Puro) at *Xho*I and *Bam*HI sites. The plasmid expressing LANA-FLAG was stored in our laboratory. The oligonucleotide sequences used in this study were shown in supplementary Table S2.

**Cell Transfection —** HEK293 and HEK293T cells were transfected with 1mg/mL polyethyleneimine (PEI) (MW40000, Yeasen Biotechnology) at a ratio of 1 μg DNA: 3 μL PEI.

**Generation of Cell Lines with Stable Expression —** Stable cell lines derived from HEK293, HEK293T, MM, KMM, and HeLa cells were established by lentiviral infection. Briefly, cells were co-transfected with lentiviral packaging plasmids (psPAX2 and VSVG) to generate lentiviruses using the PEI 4000 method with the expression plasmid pLVX-YFP vector containing AURKA^WT^, AURKA^D132A^, AURKA^1-132^ and AURKA^133-end^. Supernatants containing lentivirus were collected 48 h post-transfection, filtered, and supplemented with 6 μg/mL polybrene. These packaged viruses were used to infect cells for another 48 h, positive cells were selected with puromycin for 2 weeks, and immunoblot analysis verified protein expression. For knockout cell lines, the sequences of single-guide RNA (sgRNA) for casp3^KO^, casp6^KO^, casp7^KO^, and casp8^KO^ were designed using the online tool CRISPR Design (http://tools.genome-engineering.org) 35. Each gene was designed with 2 targets of gRNA sequences, and specific sgRNA for caspase was synthesized, annealed, and cloned into the lentiCRISPR v2 vector at its* Bsm*BI sites as previously described. The detailed gRNA target sequences are listed below. These plasmids were then transfected into HEK293T cells for 48 h, then the cells were selected with 2 μg/mL puromycin.

**RNA-Seq Analysis —** Total RNA from HeLa-vector, HeLa-AURKA^WT^, and HeLa-AURKA^133-end^ cells was extracted with Trizol (Invitrogen) according to the manufacturer's protocol, and the quality of isolated RNA was measured using NanoDrop 2000 spectrophotometer (Thermo Scientific). These RNA samples were then subjected to library preparation and sequencing by Personalbio company (Shanghai, China). The cDNA libraries were sequenced on an Illumina NovaSeq platform, removing adapter sequences and low-quality reads. The remaining high-quality reads were aligned to the human reference genome (GRCh38) by HISAT2 (http://ccb.jhu.edu/software /hisat2/index.shtml). The expression levels of each gene were normalized and calculated as the value of fragments per transcript kilobase per million fragments mapped (FPKM), which eliminates the influence of different gene lengths and sequencing discrepancies. Significantly differentially expressed genes (DEGs) (|log2FoldChange|>1, *P*-value<0.05) were assessed using DESeq2. In addition, Gene Ontology (GO) and Kyoto Encyclopedia of Genes and Genomes (KEGG) enrichment analyses of DEGs were performed using the cluster profiler software package.

**Quantitative real-time PCR —** Total RNA was isolated from cells using Trizol (Invitrogen) and reverse transcribed to cDNA with Hifair® Ⅲ 1st Strand cDNA Synthesis SuperMix Kit (Yeasen) according to the manufacturer's protocol. Real-time qPCR was performed with Hieff® qPCR SYBR Green Master Mix and the relative expression levels were calculated with the comparative threshold cycle (∆∆CT) method. Data shown are the relative abundance of the indicated mRNA normalized to that of β-actin.

**Flow Cytometric Analysis of Apoptotic Cells —** According to the manufacturer's instructions, apoptotic cells were detected using an Annexin V-PE/7-AAD apoptosis detection kit (Vazyme). Briefly, cells were collected and washed twice with PBS, suspended in the binding buffer. The cells were then incubated with Annexin V-PE and 7-AAD in the dark for 10 min at room temperature. The stained cells were analyzed in the flow cytometer BD FACS Calibur (BD Bioscience) and data were analyzed using FlowJo.

**Immunoblotting —** Cells were lysed in lysis buffer (150 mM NaCl, 50 mM Tris-HCl pH 7.5, 2 mM EDTA, 1% NP-40) containing protease inhibitor for 30 min on ice. Samples were centrifuged at 12,000 × *g* at 4 °C for 5 min and the supernatant boiled in SDS loading buffer. Equal amounts of protein were separated by SDS-PAGE and transferred to a nitrocellulose membrane. The membranes were blocked in 5% skimmed milk in PBS for 1 h and then incubated with the primary antibodies overnight at 4 °C. After 3 times washed with TBST, the blots were incubated with secondary antibodies for 1 h at room temperature. The immunoblots were detected with an Odyssey Infrared Imaging System (Li-Cor Biosciences).

**Immunofluorescence Analysis —** Transfected HEK293T cells were washed in PBS, fixed in 4% PFA, blocked with PBS containing 3% BSA and 0.5% Triton X-100 for 15 min, and incubated with the primary antibodies for 1 h at room temperature. Subsequently, the cells were washed three times with PBS and incubated with a secondary antibody (goat & Rabbit 594) for 1 h at room temperature. Nuclei were stained with 0.5 μg/mL DAPI, and coverslips were mounted with mounting medium. Cells in mitosis with centrosome and spindle formation were visualized with Leica SP8 confocal microscope.

**Live Cell Imaging —** To visualize division patterns of cells expressing AURKA and its mutants in living cells, HEK 293T cells were seeded into 4-chamber Glass Bottom Dishes (In vitro scientific) and transfected with GFP-AURKA or GFP-AURKA mutants, respectively. At 24 h post-transfection, the cell culture medium was removed and replaced with warm DMEM with 6% FBS in the incubator for 1 h. The cells were then scanned under the laser scanning confocal microscope using the Delta Vision high-resolution cell imaging systems (GE company). The chamber was maintained at 37ºC and supplemented with 5% CO_2_ with a microscope stage heater. A Zeiss 710 confocal microscope (Carl Zeiss, GERMANY) was used with 63x objectives. The data were analyzed by softWorxExplore software.

**Colony Formation Assay —** Stable HeLa and KMM cells expressing AURKA and its mutant were digested and plated in 3,000 or 10,000 cells per 10 cm^2^ dish. The cells were thoroughly mixed in 6% FBS DMEM containing 1 μg/mL puromycin and incubated at 37˚C in a humidified environmental incubator with 5% CO_2_. The medium was replaced with fresh medium every 3 days until the cultured cells had formed visible cell colonies (>50 cells/colony). After 10 days of growth, the medium was discarded and the cells were fixed with 4% paraformaldehyde for 20 min at room temperature, and then stained with 0.1% Crystal Violet Stain Solution (0.1%) for 20 min at room temperature. Colony formation in each dish was scanned by Canoscan LIDE 210.

**Tumor xenograft —** Five-week-old female nude (BALB/c) mice were used in the experiments. HeLa-Vector, HeLa-AURKA^WT,^ and HeLa-AURKA^D132A^ cells were injected into the mice subcutaneously to generate the mouse models. In total, Hela stable cells (10×10^6^) in 200 μL PBS mixed with High Concentration Matrigel (BD Biosciences) were injected subcutaneously into the flank region of nude mice. The HeLa tumor-bearing mice were divided into six groups (n=5) as follows: vector-control, vector-Taxol, AURKA^WT^-control, AURKA^WT^-Taxol, AURKA^D132A^-control, AURKA^D132A^-Taxol. The mice were injected with 200 μL saline or Taxol (10 mg/kg) every two days five times. At the end of the experiments, the tumors were subcutaneously harvested following animal sacrifice by cervical dislocation.

**Hematoxylin-Eosin Staining Assay —** Paraformaldehyde-fixed tumors were embedded in paraffin. Then the paraffin sections were deparaffinized by elution through a gradient of xylene and ethanol. The nucleus and cytoplasm of the cells were stained with hematoxylin and eosin respectively and the sections dehydrated in progressively higher concentrations of ethanol and xylene. All experiments were performed by Servicebio Company.

**TdT-mediated dUTP Nick-End Labeling (TUNEL) staining —** We utilized TUNEL assay kit (Servicebio) to detect apoptotic cells in Hela subcutaneous xenografts that were treated with or without Taxol. Apoptotic cells were determined by (DAB) staining. DAB staining resulted in blue nuclei for normal cells and yellow-brown nuclei for apoptotic cells. The apoptotic index value was obtained by dividing the number of apoptotic cells by the total number of cells in each field of view (scale bar, 100 μm). The quantification of apoptotic cells was analyzed with ImageJ software.

## Results

### AURKA cleavage at Asp^132^ is induced by oncovirus KSHV or apoptosis stimuli

**Figure 1:**
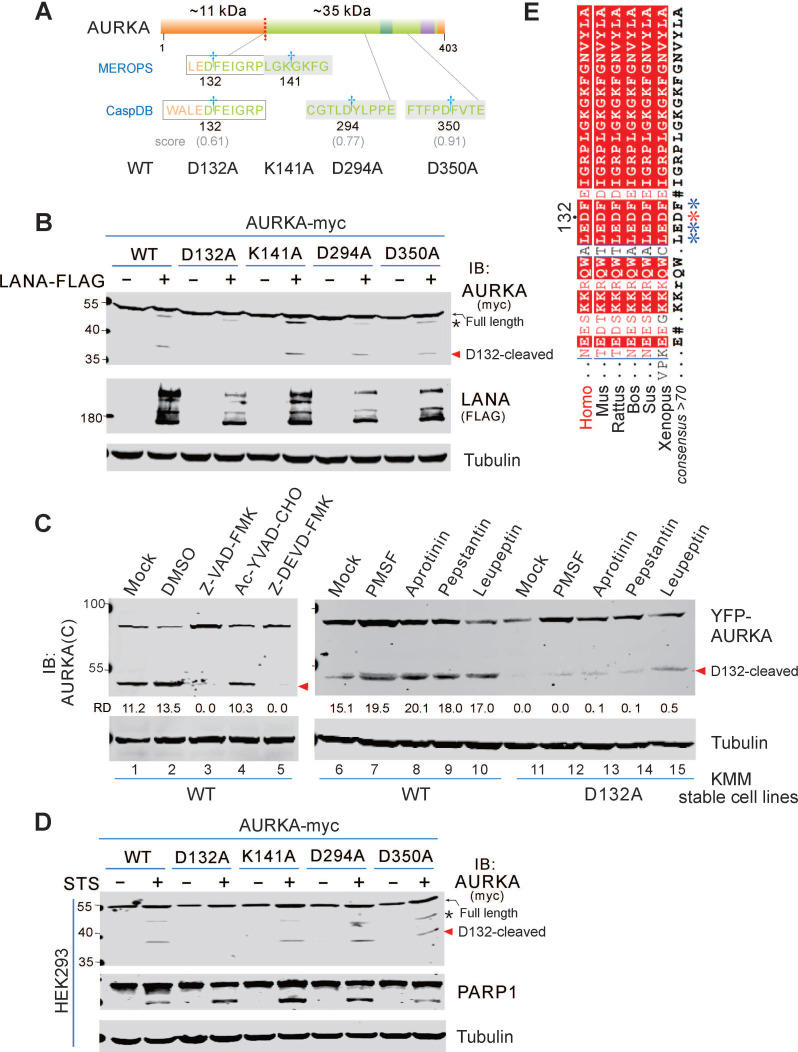
** AURKA cleavage at Asp^132^ is induced by oncovirus KSHV and apoptosis.** (**A**) Schematic of cleavage sites in AURKA predicted by online database analysis. The star indicates potential cleavage sites identified using the MEROPS and CaspDB databases. The regulatory domain (orange), catalytic domain (green), activation loop (dark green), and D-box domain (purple) of AURKA and its mutants are indicated. (**B**) Mutation of the Asp^132^ site abolishes LANA-induced AURKA cleavage. HEK293 cells were transfected with AURKA-myc or the mutants in the presence or absence of LANA-FLAG. At 48 h post-transfection, cells were harvested and whole cell lysates were subjected to immunoblotting with the indicated antibodies. (**C**) KSHV-induced AURKA^D132^ cleavage is blocked by inhibitors of pan-caspase and caspase 3, but not serine protease inhibitors. Equal numbers of KSHV-positive KMM cells stably expressing wild-type (WT) YFP-AURKA, or the D132A mutant were untreated (Mock) or treated with DMSO, pan-caspase (Z-VAD-FMK, 25 μM), caspase 1 (Ac-YVAD-CHO, 25 μM), or caspase 3 (Z-DEVD-FMK, 25 μM) inhibitor for 24 h, or different protease inhibitor for 6 h, followed by immunoblotting analysis using the antibody against AURKA(C). (**D**) Mutation of the D132 site blocks staurosporine (STS)-induced AURKA cleavage. Equal numbers of HEK293 cells were transiently transfected with AURKA-myc or its mutants as indicated for 24 h, then treated with or without STS (1 μM) for 6 h, followed by immunoblotting with indicated antibodies (myc-tag antibodies were used to detect exogenous AURKA). The arrow indicates the Asp^132^-cleaved product of AURKA. The asterisk indicates uncharacterized bands. (**E**) Alignment of the ^129^ALEDFE^134^ sequence of AURKA from different species. The proposed caspase cleavage site is indicated by an asterisk.

Our previous study revealed that viral-mediated AURKB is cleaved by a serine protease at Asp^76^ to generate a 35 kDa fragment (aa 77-344), which promotes cell segregation and tumorigenesis 34. However, the cleavage of AURKA and the related molecular mechanisms remain unknown. Two potential cleavage sites were identified in AURKA and AURKB based on the MEROPS database online analysis. The AURKA cleavage sites include Asp^132^ (corresponding to Asp^76^ in AURKB) and Lys^141^ (corresponding to Lys^85^ in AURKB). The amino acid sequence of AURKA was also analyzed for caspase-mediated cleavage sites using the CaspDB database 36. Cleavage at Asp^132^ of AURKA by caspase was predicted with a 0.60 probability score, and cleavage at Asp^350^ and Asp^294^ in the C-terminal region of AURKA by caspase was predicted with probability scores of 0.91 and 0.77, respectively (Figure 1A). Based on the predictions of MEROPS and CaspDB databases, we speculated that Asp^132^ was a physiological cleavage site of AURKA. Thus, we generated AURKA with a myc-tag at the carboxyl terminus (AURKA-myc) and substituted aspartic (D) or arginine (K) amino acids with alanine (A) to generate 4 AURKA mutants, including D132A, K141A, D294A, and D350A.

We previously demonstrated that the KSHV latent antigen LANA interacts with AURKA and pulls down a smaller protein band (∼35 kDa) 34. To determine if LANA induces AURKA cleavage at the Asp^132^ residue, we transfected exogenous AURKA-myc or the point mutants into HEK293 cells in the presence or absence of LANA-Flag, followed by immunoblotting with anti-myc antibody. Consistent with our previous findings, we detected a 35 kDa band for AURKA in cells co-transfected with LANA (Figure 1B). The myc-tag of AURKA is located at the C terminus; thus, the AURKA 35-kDa fragment is a short form of the C terminus. As expected, the alanine substitution of D132, but not the K141, D294, or D350 substitutions, was resistant to LANA-induced AURKA cleavage (Figure 1B). To confirm this cleavage under physiological conditions, we generated KMM (KSHV-infected rat endothelial cells) stably expressing yellow fluorescent protein (YFP)-tagged wild-type AURKA or its D132A mutant using a lentiviral system, followed by immunoblotting with AURKA(C) antibody (C-terminal antibody), which recognizes the 350-450 aa region of human AURKA (Figure S1A). Immunoblotting with a GFP antibody showed that the D132A mutation was resistant to AURKA proteolysis, whereas wild-type AURKA was cleaved (Figure S1B). We attempted to construct BCBL1 (KSHV-infected human B lymphoma) cells stably expressing AURKA or the mutants, unfortunately, several trials with long-term puromycin selection failed. However, BCBL1 cells were infected with lentivirus carrying YFP-tagged AURKA^WT^, AURKA^D132A^, or cleaved (1-132 or 133-end) mutants for 48 h, cleaved bands (the same size as the 1-132 or 133-end cleaved products) were detected in cells transfected with AURKA^WT^ but not AURKA^D132A^ (Figure S1B).

To identify the protease that cleaves AURKA at Asp^132^ in the KSHV-infected cells, we treated KMM cells stably expressing either YFP-AURKA^WT^ or AURKA^D132A^ with different inhibitors of serine protease, aspartyl protease, or serine/cysteine/threonine proteases for 6 h. Unlike the serine protease-dependent cleavage of AURKB induced by KSHV, these inhibitors did not efficiently block AURKA cleavage (Figure 1C). These results indicate that cleavage of AURKA at Asp^132^ did not rely on serine proteases. Since the Asp^132^ of AURKA was also a potential caspase cleavage site based on the CaspDB database analysis, KMM cells stably expressing YFP-tagged AURKA^WT^ or AURKA^D132A^ were treated with the inhibitors of pan-caspase (Z-VAD-FMK), caspase 1 (Ac-YVAD-CHO), and caspase 3 (Z-DEVD-FMK). Both pan-caspase and caspase 3 inhibitors, but not the caspase 1 inhibitor, completely blocked the cleavage of AURKA at Asp^132^ (Figure 1C, left panel). These results indicate that the Asp^132^-cleavage of AURKA is mediated by caspase 3.

Caspase 3 is an apoptotic executioner caspase, which cleaves many substrate proteins. The caspase 3 inhibitor prevents AURKA cleavage. Thus, we hypothesized that AURKA is cleaved at Asp132 during apoptosis. To test this hypothesis, we transiently transfected HEK293 cells with AURKA-myc or the point mutants (including D132A, K141A, D294A, and D350A) in the presence or absence of staurosporine (STS, a potent apoptotic stimulus in many cell lines) 37-39. AURKA cleavage was monitored by immunoblotting with the anti-myc antibody. STS treatment strongly induced AURKA cleavage in HEK293 cells, but no cleavage was detected in the AURKA^D132A^ mutant (Figure 1D), This result indicates that Asp^132^-cleavage of AURKA is induced during apoptosis. Interestingly, we found the Asp132 residue is highly conserved in AURKA gene from different species (Figure 1E), indicating that Asp132-cleavage of AURKA may be a common response to cell stress.

### Asp^132^-cleavage of AURKA occurs in various cancer cells during apoptosis

STS plays an important role in successful anti-cancer drugs 40. To verify the effect of STS-induced apoptosis, both KSHV-infected BCBL1 and uninfected BJAB cells were treated with different concentrations of STS for 3 h, followed by flow cytometry analysis of apoptotic cells. Low concentration (1 μM) of STS efficiently induced over 70% apoptosis in BCBL1 cells but induced only 17.5% apoptosis in BJAB cells (supplementary Figure S2), suggesting that KSHV-infected cells are more sensitive to STS than uninfected cells.

**Figure 2:**
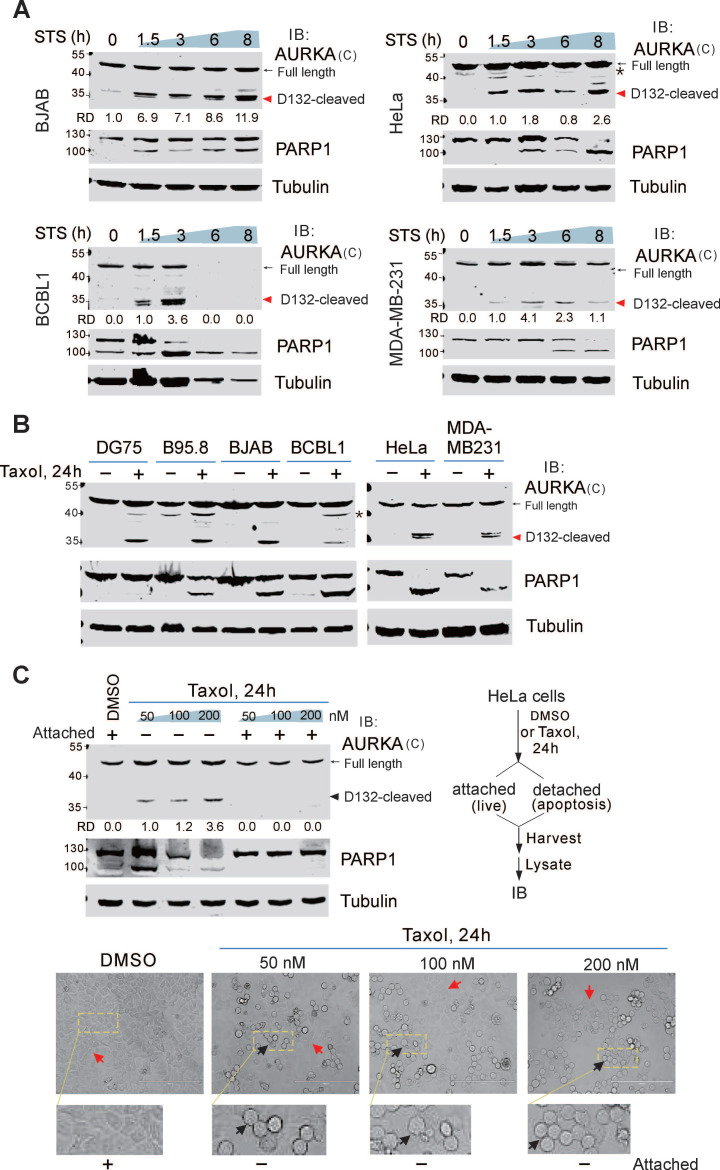
** Asp^132^ cleavage of AURKA commonly occurs in cancer cells with apoptosis.** (**A**) Asp**^132^** cleavage of AURKA occurs in different cancer cell types during staurosporine (STS)-mediated apoptosis. Equal numbers of cells were untreated or treated with STS (1 μM) for the indicated times, followed by immunoblotting analysis with antibodies as indicated. The arrow indicates the Asp^132^-cleavage product of AURKA. The asterisk indicates uncharacterized bands. (**B**) AURKA undergoes Asp^132^ cleavage in cancer cells after Taxol-induced apoptosis. Equal numbers of cells were stimulated with or without 100 nM Taxol for 24 h, followed by immunoblotting analysis with the indicated antibodies. (**C**) AURKA Asp^132^ cleavage does not occur in Taxol-resistant cancer cells. Equal numbers of HeLa cells were treated with different concentrations (0, 50, 100, and 200 nM) of Taxol. At 24 h post-treatment, the attached (live) and detached (apoptosis) cells were subjected to immunoblotting analysis with antibodies as indicated. The representative images of cells with or without Taxol treatment are shown at the bottom panels.

To determine if cleavage of endogenous AURKA is associated with apoptosis, both BCBL1 and BJAB cells were treated with or without STS and the apoptotic marker PARP1 was detected by immunoblotting. In addition to the full-length AURKA, a ~35 kDa band (D132-cleaved product of C-terminus) was detected in both BCBL1 and BJAB cells after STS treatment using the antibody specific for the C-terminal region of AURKA (residues 350-403 amino acid) (Figure 2A, supplementary Figure S1A). In BJAB cells, the cleavage of AURKA at Asp^132^ was detected after 1.5 h of STS incubation, and peaked after 8 h. In BCBL1 cells, the cleavage was observed after 1.5 h of STS treatment, peaked at 3 h, and was completely degraded after 6 h. This suggests that Asp^132^-cleavage of AURKA occurs at an early stage of apoptosis. To investigate whether Asp^132^-cleavage of AURKA in STS-induced cell apoptosis is affected by viral infection, we examined the cleavage of AURKA in both non-viral (DG75 and iSLK) and viral (BC3, B95.8, and iSLK-16) cancer cells treated with STS at different time points. Asp^132^-cleavage of AURKA was observed in all cells following apoptotic stimuli (supplementary Figure S3A-B), indicating that cleavage of AURKA at Asp^132^ during STS-induced apoptosis does not depend on viral infection.

To further demonstrate whether the Asp^132^-cleavage of AURKA is cell-type dependent, we measured cleavage in various types of cancer cells, including cervical cancer cells (HeLa), breast cancer cells (MDA-MB-231), and liver cancer cells (SMMC-7721 and HLE). Asp^132^-cleavage of AURKA occurred in all cell types upon STS treatment (Figure 2A-B; supplementary Figure S3C). These findings strongly support the notion that Asp^132^-cleavage of AURKA is a common event during STS-induced apoptosis*.*

To determine if Asp^132^-cleavage of AURKA is triggered by other apoptotic stimuli, we induced apoptosis with a common chemotherapeutic drug paclitaxel (Taxol) in different cancer cell lines, including primary effusion lymphoma, cervical, and breast cancers. Asp^132^-cleavage of AURKA was detected in all cell types upon Taxol treatment (Figure 2B), suggesting that Asp^132^-cleavage of AURKA is involved in Taxol induction of apoptosis. Unlike STS treatment, when apoptosis was induced in HeLa cells, with Taxol, not all cells underwent apoptosis (detached and rounded morphology) (Figure 2C, bottom panel). To verify this phenomenon, we treated HeLa cells with increasing concentrations of Taxol and harvested the attached (live) and detached (apoptosis) cells. Asp^132^-cleavage of AURKA was detected in apoptotic cells, but not in live cells (Figure 2C), supporting the role of Asp^132^-cleavage of AURKA in Taxol-mediated apoptosis.

### Cleavage of AURKA at Asp^132^ relies on caspases 3/7/8

**Figure 3:**
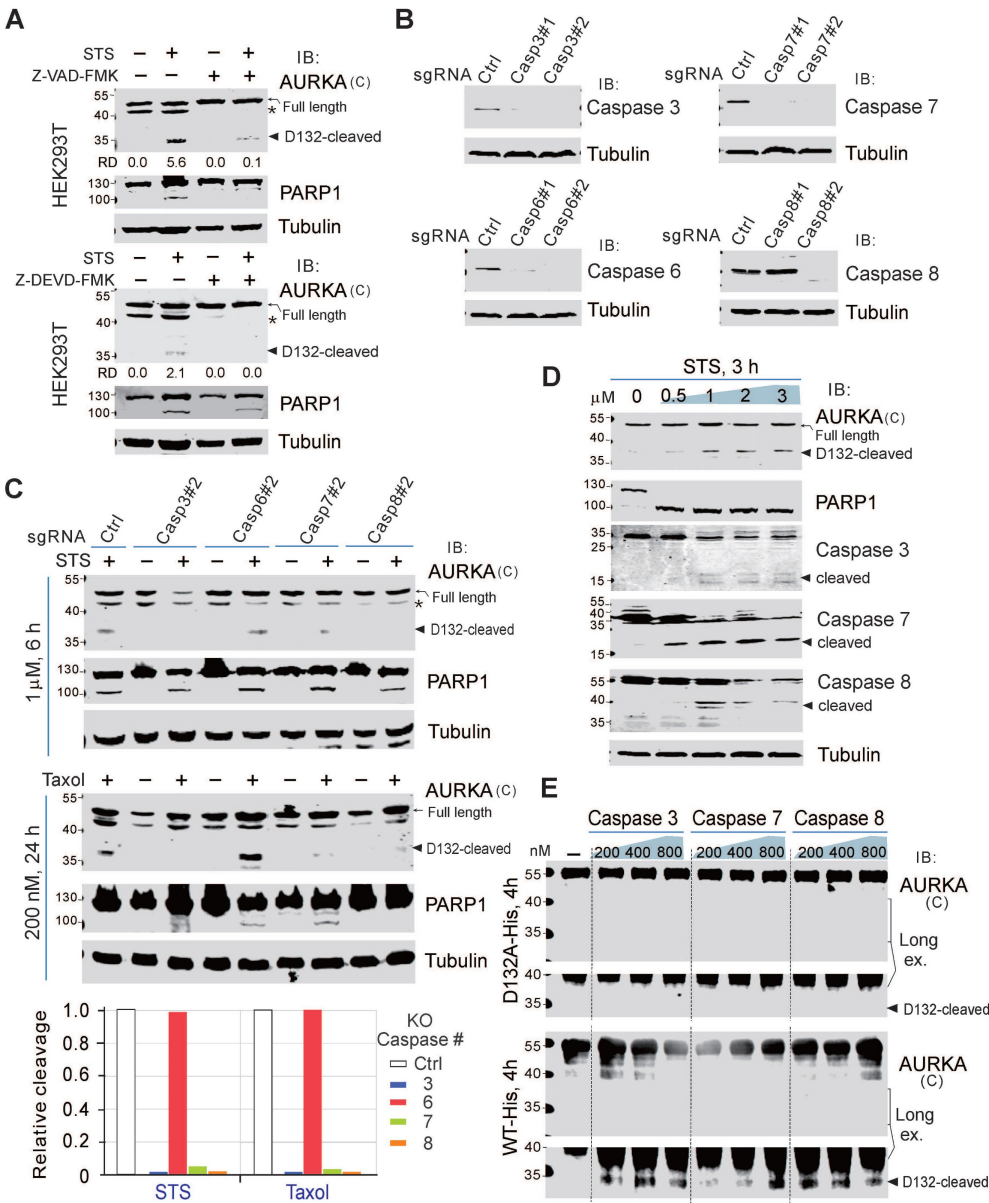
** Asp^132^-cleavage of AURKA depends on Caspase 3/7/8.** (**A**) The Asp^132^ cleavage of AURKA induced by STS is blocked by the pan-caspase inhibitor (Z-VAD-FMK) or caspase 3/7 inhibitor (Z-DEVD-FMK). Equal numbers of HEK293T cells were preincubated with Z-VAD-FMK (25 μM) or Z-DEVD-FMK for 1 h, and then treated with staurosporine (STS, 1 μM) for 6 h, followed by immunoblotting analysis with the indicated antibodies. The asterisk indicates uncharacterized bands. (**B**) The knockout efficiency of caspase 3, 6, 7, and 8 was verified by immunoblotting analysis in HEK293T cells. Tubulin was used as a control. (**C**) Knockout of caspase 3/7/8 consistently blocks the STS-mediated Asp^132^ cleavage of AURKA. Equal numbers of HEK293T parental and knockout (caspases 3, 6, 7, or 8) cells were treated with STS or Taxol, followed by immunoblotting with the indicated antibodies. The arrow indicates the Asp^132^-cleaved product of AURKA. Relative efficiency of STS/Taxol-mediated Asp^132^ cleavage of AURKA in the presence or absence of caspase 3, 6, 7, and 8 as shown in the bottom panel. (**D**) The levels of Asp^132^ cleavage of AURKA are consistent with activation of cleaved Caspase 3, 7, and 8 in BCBL1 cells after STS treatment. Equal numbers of BCBL1 cells were treated with increasing concentrations (0, 0.5, 1, 2, and 3 μM) of STS for 3 h, followed by immunoblotting with the indicated antibodies. The arrow indicates the Asp^132^-cleaved product of AURKA or cleaved active forms of the caspase. (**E**) Caspases 3/7/8 cleave AURKA-His at Asp^132^* in vitro*. Purified AURKA-His protein was treated with different doses (0.2, 0.4, and 0.8 μM) of caspases for 4 h at 37ºC and analyzed by immunoblotting as indicated.

The activation of caspases is a key event in apoptosis. To confirm the involvement of caspases in the cleavage of AURKA at Asp^132^, we pretreated HEK293T and BJAB cells with a pan-caspase inhibitor (Z-VAD-FMK) for 1 h, before treating the cells with STS for 6 h. AURKA cleavage at Asp^132^ was dramatically inhibited by the caspase inhibitor, even after treatment with STS (Figure 3A; supplementary Figure S4), suggesting that the proteolytic cleavage of AURKA is dependent on the activation of caspase. The caspase-3 inhibitor (Z-DEVD-FMK) also prevented AURKA cleavage in HEK293T cells during STS-induced apoptosis (Figure 3A, bottom panel), indicating that caspase 3 may be a key protease for inducing AURKA cleavage in apoptosis. In addition to caspase 3, Z-DEVD-FMK inhibits caspase 6, 7 and 8 41. To determine the specific caspase involved in Asp^132^-cleavage of AURKA during apoptosis, we generated caspases 3, 6, 7, and 8-knockout HEK293T cells using the CRISPR-Cas9 system (Figure 3B). The knockout cells were treated with or without STS. Interestingly, knockout of caspase 3, 7, or 8 almost eliminated Asp^132^-cleavage of AURKA compared with the parental control cells, whereas caspase 6 deficiency did not affect AURKA cleavage in response to STS treatment (Figure 3C, upper panels). Similar results were observed with Taxol treatment (Figure 3C, lower panels). These results indicate that caspases 3, 7, and 8, but not 6, mediate AURKA cleavage at Asp^132^ in response to apoptotic stimuli. In agreement with this observation, the activities of caspases 3, 7, and 8 were associated with Asp^132^-cleavage of AURKA in BCBL1 cells after treatment with increasing concentrations of STS (Figure 3D). In the protein cleavage *in-vitro* assay, Asp^132^ cleavage of the wild-type AURKA, but not the D132A mutant, was detected after co-incubation with caspase 3, 7, or 8 *in vitro* (Figure 3E). These results indicate that caspase 3, 7, and 8 are involved in the Asp^132^-cleavage of AURKA during apoptosis.

The cleavage site at Asp^132^ of AURKA was highly conserved in six different species (Figure 1E). Thus, the role of caspase 3/7/8-mediated AURKA cleavage at Asp^132^ in cells undergoing apoptosis should be important. Gene mutations commonly occur in drug-resistant tumor cells, leading to chemotherapy failure 42. The alteration frequency of AURKA correlated with caspase 3, 7, and 8 in the TCGA Pan-Cancer Atlas study (supplementary Figure S5). Interestingly, among the mutation sites of AURKA in 10,967 tumor samples from 21 types of tumors, the amplification of AURKA was highly associated with mutations of caspases 3, 7, and 8, indicating the biological significance of the caspase 3/7/8-mediated Asp^132^ cleavage of AURKA in chemotherapy.

### Asp^132^-cleaved product of AURKA elicits cell apoptosis

**Figure 4:**
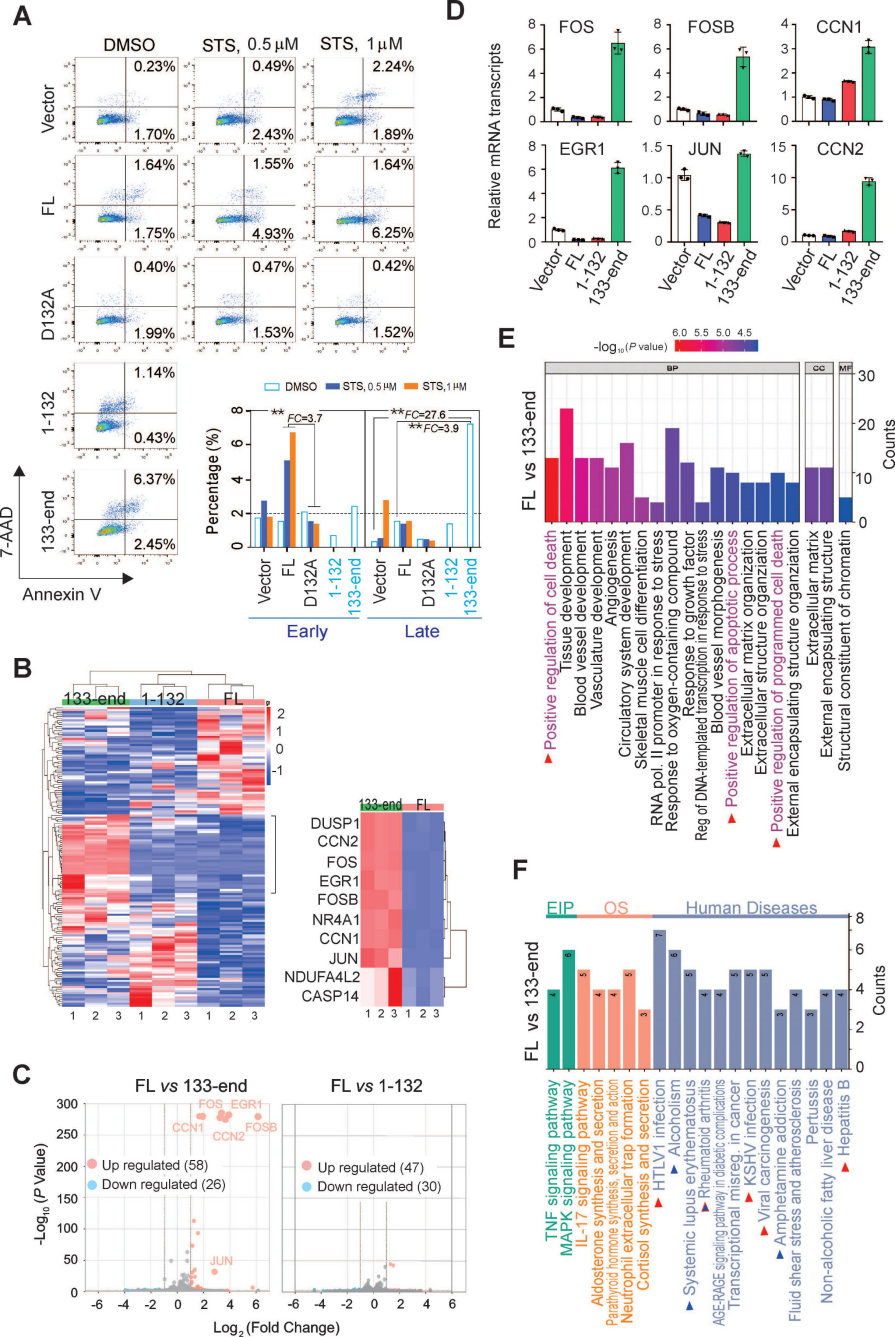
** Asp^132^ cleavage of AURKA induces cell apoptosis.** (**A**) Mutation of the AURKA^D132^ cleavage site prevents from STS-induced apoptosis. Equal numbers of HEK293 cells stably expressing YFP-tagged full-length (FL) AURKA or the D132A, 1-132, and 133-end mutants or vector only were individually treated with DMSO or STS (0.5 or 1 μM) for 3 h, followed by staining with Annexin V-PE/7-AAD and flow cytometry analysis. (**B**) Hierarchical clustering analysis of global gene expression in HeLa cells with YFP-tagged full-length AURKA or the N-cleaved (1-132) or C-cleaved (133-end) mutants. Deep RNA-seq analyses were repeated three times. The heatmap of the top 10 upregulated genes in the AURKA^FL^ and AURKA^133-end^ groups are highlighted in the right panel. (**C**) Volcano plot of differentially expressed genes (DEGs) in the AURKA^FL^ *vs.* AURKA^1-132^ and AURKA^FL^ *vs.* AURKA^133-end^ groups from *panel B*. The number of up-regulated (orange) and down-regulated (blue) genes are shown. The grey dots indicate genes that are not significantly different. Log2(Fold Change) >1 and *P*-value <0.05 were set as a cut-off. (**D**) Validation of the top 6 genes related to AURKA^133-end^ from *panel C* using quantitative PCR. AURKA^133-end^ upregulates relative transcript levels of AP-1 members, including JUN, FOS, and FOSB, and the downstream EGR1, CCN1 and CCN2. (**E**) GO functional enrichment analysis of DEGs between the AURKA^FL^ and AURKA^133-end^ groups from *panel B*. (**F**) KEGG enrichment analysis of DEGs from AURKA^FL^ *vs.* AURKA^133-end^ based on the RNA-Seq data from *panel B*. The arrows highlight the human diseases related to C-terminal product of AURKA cleavage at Asp^132^.

The Asp^132^-cleavage of AURKA is induced by caspases 3, 7, and 8, and is activated during apoptosis. Thus, the role of the Asp^132^-cleavage product of AURKA in the process of cell apoptosis was investigated. HEK293 cells stably expressing YFP-tagged wild-type AURKA and the uncleavable D132A, 1-132 (N-cleaved), and 133-end (C-cleaved) mutants were treated with different concentrations of STS, followed by cell cytometry with Annexin-PE/7-VAD staining for apoptosis. The mutation of AURKA^D132A^ efficiently blocked early apoptosis induced by STS compared with wild-type AURKA^FL^ cells (Figure 4A). Moreover, the C-cleaved products of AURKA significantly induced cell late apoptosis (6.37%) compared with both the wild-type (1.64%) and vector control (0.23%) groups. These findings confirm the role of caspase-mediated cleavage of AURKA at Asp^132^ in eliciting cell apoptosis.

To gain insight into the molecular mechanisms underlying AURKA cleavage at Asp^132^ during apoptosis, RNA deep-sequencing of HeLa cells stably expressing YFP-AURKA^FL^, YFP-AURKA^1-132,^ and YFP-AURKA^133-end^ was performed (Supplementary Figure S6A, Figure 4B). The clustering analysis showed different transcript profiles for cells expressing AURKA^FL^ and AURKA^1-132^ or AURKA^133-end^ (Figure 4B). Seventy-seven genes (47 upregulated and 30 downregulated) in the AURKA^1-132^ group and 84 (58 upregulated and 26 downregulated) genes in the AURKA^133-end^ group were differentially expressed compared with AURKA^WT^ cells (Figure 4C). The top 10 up-regulated genes in the AURKA^133-end^ group are components of AP-1 and are associated with the regulation of cell death and apoptosis (Figure 4B right panel). To validate the reliability of the RNA-Seq data, the upregulation of six representative genes associated with cell death and apoptosis, including Fos, FosB, Jun, EGR1, CCN1, and CCN2, in the presence of AURKA^133-end^ were confirmed by quantitative PCR (Figure 4D).

To determine the biological significance of the differentially expressed genes, gene ontology functional classification was conducted. The differentially-expressed genes from the AURKA^133-end^ group, but not the AURKA^1-132^ group, were exclusively enriched in signaling pathways related to the positive regulation cell death and apoptosis (Figure 4E; Figure S6B). Intriguingly, KEGG enrichment analysis revealed that the differentially-expressed genes from the AURKA^133-end^ but not the AURKA^1-132^ group were tightly associated with viral carcinogenesis and oncogenic virus infection, including HTLV1 and KSHV. This result is consistent with the observation that LANA induces Asp^132^-cleavage of AURKA (Figure 4F, supplementary Figure S6C). These findings collectively indicate that Asp^132^-cleavage of AURKA plays a crucial role in inducing apoptosis by upregulating multiple pro-apoptotic molecules.

### Asp^132^-cleavage product of AURKA disrupts centrosome formation and spindle assembly

**Figure 5:**
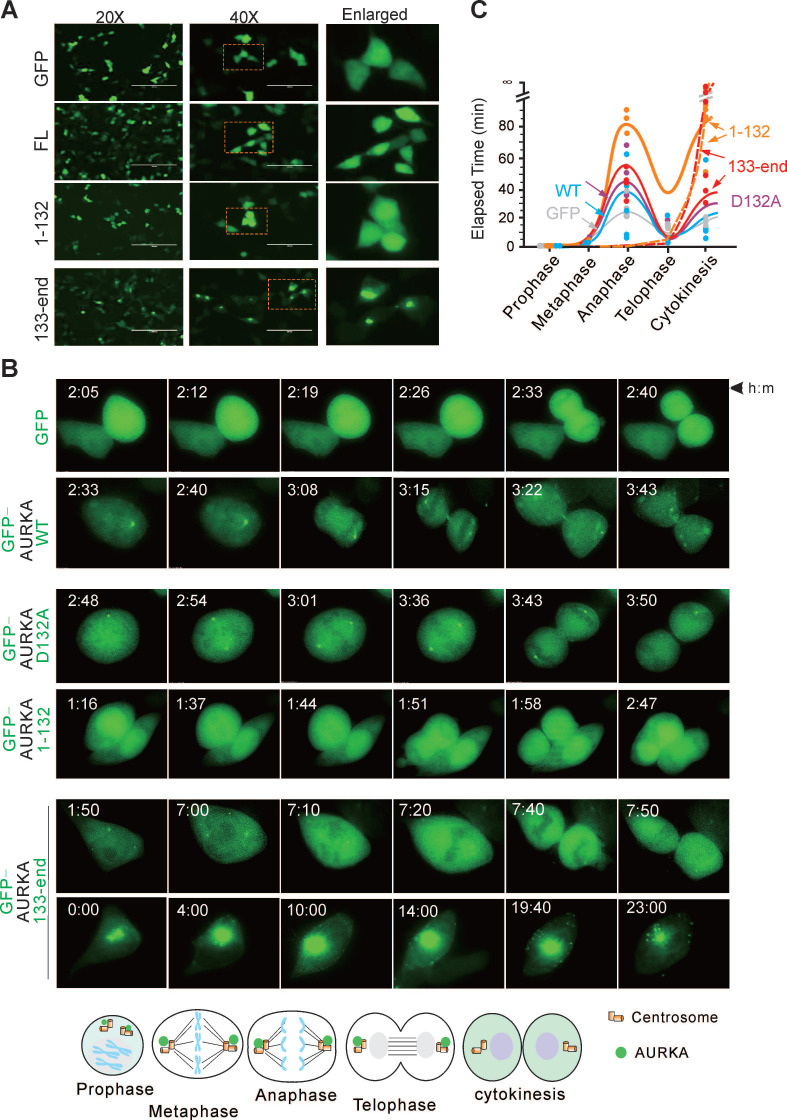
** Asp^132^ cleavage of AURKA disrupts cell mitotic progression.** (**A**) Representative micrographs show the localization of Asp^132^-cleaved products of AURKA in HEK293T cells. FL, full-length. (**B**) Cell cycle progression is disrupted by the Asp^132^-cleaved products of AURKA. Live cell imaging of HEK293T cells transiently transfected with GFP tagged-AURKA^WT^ or the cleaved products GFP-AURKA^1-132^ and GFP-AURKA^133-end^ for 24 h. Living cells were monitored using time-lapse fluorescence microscopy. Bottom panel, schematic of localization of GFP-AURKA in mitotic cells. **(C)** Quantitative analysis of the elapsed time from prophase to cytokinesis of mitotic cells expressing different AURKA mutants from panel B. Cells were classified by chromosome and cell morphology.

To determine how the Asp^132^-cleaved product of AURKA induces cell apoptosis, we examined the subcellular localization of the Asp^132^-cleavage products of AURKA in HEK293T cells transiently expressing GFP-tagged wild-type AURKA and the cleaved mutants using confocal microscopy analysis. The GFP-tagged AURKA displayed diffuse cytoplasmic and nuclear localization. However, the N-cleaved product of AURKA^1-132^ accumulated in the nucleus, and the C-cleaved product of AURKA^133-end^ translocated from the nucleus to the cytoplasm and often formed granular aggregates (Figure 5A).

AURKA localizes to the centrosome and mitotic spindle poles during mitosis 43. Therefore, the impact of Asp^132^-cleavage of AURKA on the cell cycle was investigated. HEK293T cells expressing wild-type GFP-tagged AURKA or the mutants were monitored by time-lapse microscopy (Figure 5B). Cells expressing the AURKA^D132A^ mutant took more time to transition from metaphase to telophase. Interestingly, cells expressing the N-cleaved product of AURKA^1-132^ were unable to form centriole-like punctate dots, and the average time was increased compared with the time in cells expressing wild-type AURKA. Notably, centriole-like punctate dots were smaller but increased in number in cells expressing the C-cleaved product of AURKA^133-end^. Additionally, overexpression of the C-cleaved product of AURKA^133-end^ results in aggregated punctate dots in the cytoplasm in a large proportion of cells, eventually leading to cell death (Figure 5B-C). These findings suggest that Asp^132^-cleavage of AURKA disrupts cell mitotic progression.

**Figure 6:**
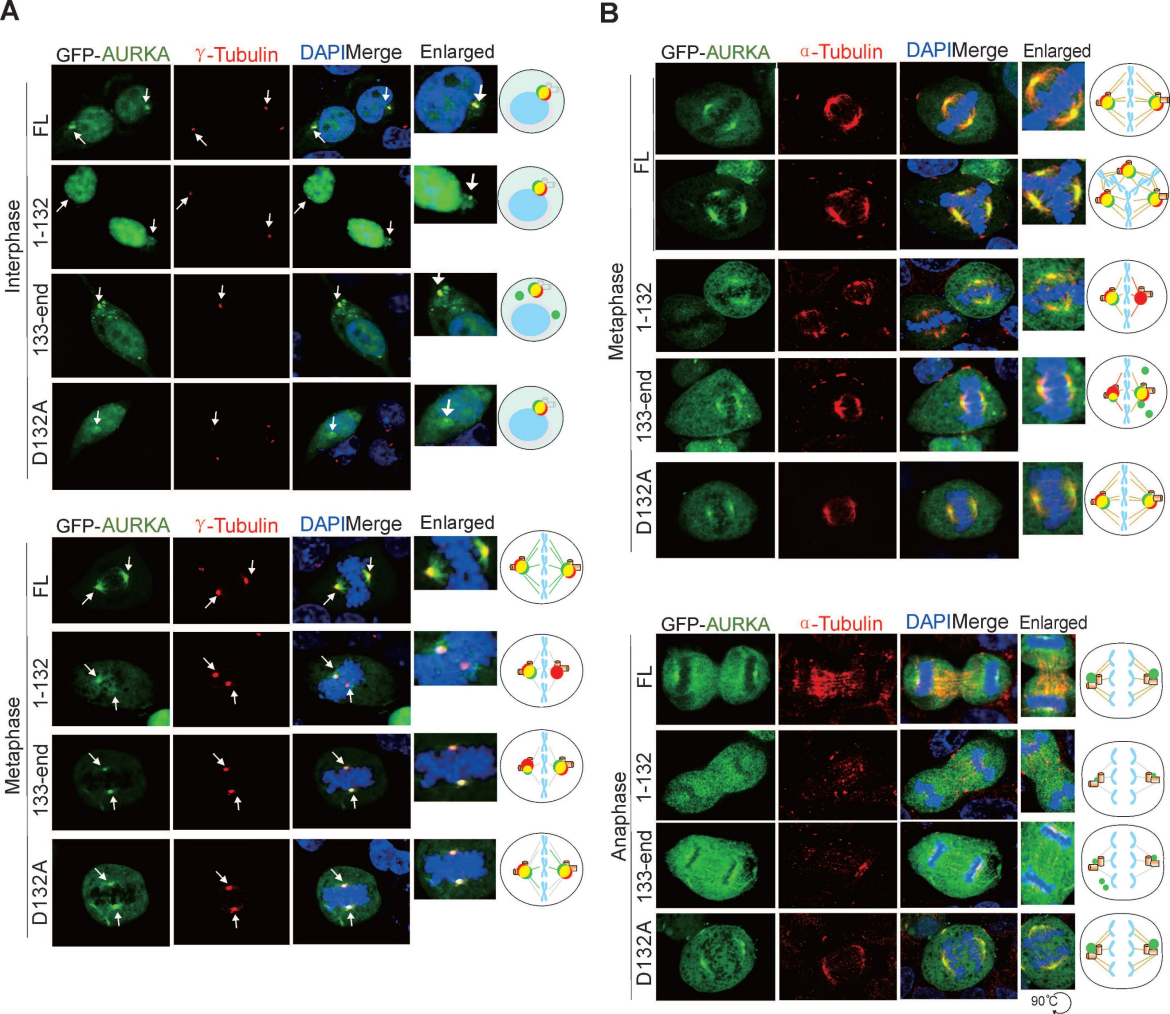
** Asp^132^-cleaved form of AURKA disrupts centrosome formation and spindle assembly.** (**A**) The Asp^132^-cleaved form of AURKA disrupts centrosome formation in mitosis. HEK293T cells were transiently transfected with full-length (FL) AURKA or the D132A, N-terminus (1-132), and C-terminus (133-end) GFP-tagged mutants. At 24 h post-transfection, immunofluorescence analysis with antibodies against γ-tubulin (red) for the centrioles and DAPI for the nuclei were carried out. Enlarged views of the centrosome are highlighted with schematics in the right panels. Scale bar, 10 μm. (**B**) Asp^132^-cleaved form of AURKA disrupts spindle assembly in mitosis. HEK293T cells were transiently transfected with full length (FL) AURKA or the D132A, N-terminus (1-132), and C-terminus (133-end) GFP-tagged mutants. At 24 h post-transfection, cells were subjected to immunofluorescence analysis with antibodies against α-tubulin (red) for mitotic spindle and DAPI (blue) for nuclei. Enlarged views of the spindles are highlighted with schematics in the right panels. Scale bar, 10 μm.

To confirm the impact of Asp^132^-cleavage of AURKA on centrosome formation, HEK293T cells transfected with GFP-tagged AURKA or the Asp^132^-cleaved or site mutants were subjected to immunofluorescent staining with γ-tubulin (a centrosome marker) antibodies. AURKA^1-132^ failed to efficiently form regular sized punctate dots of centrosomes during interphase, and exhibited only one instead of two centriole punctate dots during metaphase. In contrast, AURKA^133-end^ presented a greater number of centriole-like punctate dots, but not all punctate dots co-localized with γ-tubulin at interphase. In metaphase, one of the two punctate dots of AURKA^133-end^ was much smaller and failed to fully colocalize with endogenous γ-tubulin. Cells expressing AURKA^D132A^ were similar to cells expressing AURKA^WT^ (Figure 6A). These findings suggest that Asp^132^-cleavage of AURKA leads to defective centrosome maturation.

AURKA is required for the assembly of the bipolar spindle, which is essential for the segregation of chromosomes during cell division 44. Thus, we performed immunofluorescence staining of α-tubulin to verify the effects of Asp^132^-cleavage of AURKA on bipolar spindle assembly. Both AURKA^1-132^ and AURKA^133-end^ formed weak, sparse, or short astral microtubules during metaphase and anaphase compared with AURKA^WT^ or AURKA^D132A^ (Figure 6B). These data suggest that the Asp^132^-cleaved products of AURKA induce centrosome abnormalities and disrupt bipolar spindle formation.

### Asp^132^ mutation of AURKA abolishes the sensitivity of cancer cells to Taxol

**Figure 7:**
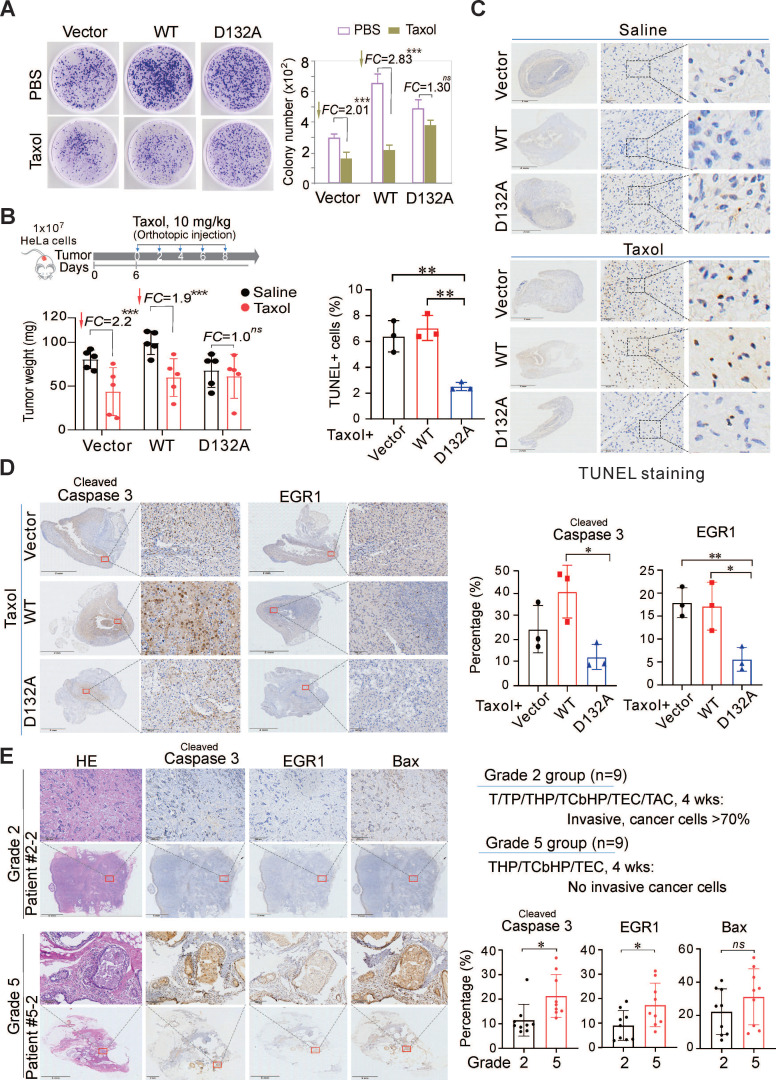
** Asp^132^ mutation of AURKA abolishes Taxol-induced apoptosis and cancer regression *in vitro* and *in vivo*.** (**A**) Taxol dramatically reduces colony formation of cells expressing wild-type AURKA but less in the D132A mutant *in vitro*. Equal numbers of HeLa cells stably expressing the vector, YFP-AURKA^WT^, or the uncleavable mutant YFP-AURKA^D132A^ were inoculated for 10 days and then treated with PBS or Taxol (25 nM) for 48 h. The relative amounts of colony formation were calculated from three independent experiments. ***, *p*<0.001. FC, fold change. (**B**) Taxol dramatically reduces the growth of tumor cells expressing wild-type AURKA but not the D132A mutant *in vivo*. Equal numbers of HeLa cells stably expressing the vector, wild-type AURKA, or the D132A mutant were mixed with Matrigel and injected subcutaneously into nude mice, followed by orthotopic administration of Taxol every two days. Mice were sacrificed on day 19, and tumor tissues (photographs shown in supplementary Figure S8A) were harvested. Tumor weights are shown in the bottom panel. ***, *p*<0.001. FC, fold change. (**C**) Taxol induces apoptosis of tumor cells expressing wild-type AURKA but not the D132A mutant*.* Tumor tissues from panel B were subjected to TUNEL staining. The percentage of TUNEL-positive cells in the Taxol-treated groups was calculated and shown in the left panel. The untreated control (Saline) groups are shown in the top panel, and the Taxol-treated groups are shown in the bottom panel. Scale bar, 50 µm, **, *p*< 0.01. (**D**) Taxol induces the expression of cleaved caspase 3 and EGR1 in tumor cells expressing wild-type AURKA but not the D132A mutant*.* The tumor tissues from Figure 7B were subjected to immunohistochemistry with the indicated antibodies, and representative images are shown on the left panel. Analysis of cleaved caspase 3 and EGR1 (Bax staining shown in supplementary Figure S8B) levels in the Taxol-treated groups. Scale bar, 50 µm, **, *p*< 0.01. (**E**) Increased expression of caspase 3 and EGR1 in the breast cancer of patients effectively treated with Taxol. The breast cancer tissues (n=18) from patients treated with different combination Taxol treatments for 4 weeks were divided into two groups of grade 2 and grade 5 (top panel) and subjected to immunohistochemistry with the indicated antibodies. Representative images of patients are shown (the enlarge view is shown on the top panels, the other is shown in supplementary Figure S9 and Table S1). The percentage of cleaved caspase 3, EGR1, or Bax-positive cells (right panel). Scale bar, 200 µm, *, *p*< 0.05. *ns*, non-significant.

Since chemotherapeutic drugs STS/Taxol could induce Asp^132^-cleavage of AURKA, and the Asp^132^-cleaved products could disrupt centrosome formation and spindle assembly for cell apoptosis, we first investigated the effects of the Asp^132^-mutation of AURKA on the cytotoxicity of chemotherapeutic drugs in HeLa cells *in vitro*. Equal numbers of HeLa cells stably expressing either AURKA^WT^, the uncleavable AURKA^D132A^ mutant, or the YFP vector alone were grown for 10 days and then treated with 25 nM Taxol for 48 h. The results showed that both AURKA^WT^ and the vector group exhibited a significant reduction (2.83-fold *vs* 2.01-fold) in colony formation in response to Taxol (Figure 7A). In contrast, the AURKA^D132A^ group did not exhibit a significant change (1.3-fold) after Taxol treatment (Figure 7A). This indicates that the Asp^132^-mutation of AURKA did decrease the sensitivity of cancer cells to Taxol *in vitro*. To further confirm the effects of Asp^132^-cleaved products of AUKRA on cell apoptosis, equal numbers of HeLa or KMM cells stably expressing AURKA^WT^, AURKA^1-132^ (N-cleaved), AURKA^133-end^ (C-cleaved), or the YFP vector alone, were subjected to colony formation assays. The Asp^132^-cleaved products of AUKRA (AURKA^1-132^ and AURKA^133-end^) did dramatically reduce colony formation in both KMM and HeLa cells *in vitro* when compared to AURKA^WT^ (supplementary Figure S7A-B). The *in vivo* role of Asp^132^-cleavage of AURKA on the anticancer efficacy of Taxol was determined in a Matrigel Plug tumor xenograft model with human cervical carcinoma HeLa cells stable expressing AURKA^WT^, AURKA^D132A^, or YFP vector in the presence or absence of Taxol for 8 days. The expression of AURKA^WT^ significantly enhanced tumor size compared to the vector alone, and a Taxol treatment reduced the tumor size in both groups (Figure 7B, supplementary Figure S8). In contrast, tumor size was not reduced in mice bearing the AURKA^D132A^ compared to AURKA^WT^ in response to Taxol (Figure 7B). These results confirmed the suppression of apoptosis by uncleaved AURKA in response to Taxol. The expression of cleaved caspase 3 and EGR1 decreased in the AURKA^D132A^ tumor tissues after Taxol treatment compared with the expression levels in the AURKA^WT^ or vector groups (Figure 7C-D). These data demonstrate that the cytotoxicity of Taxol depends on Asp^132^-cleavage of AURKA.

To determine whether the efficacy of Taxol chemotherapy in patients is related to the Asp^132^-cleavage of AURKA for apoptosis, we collected 18 primary tumor tissues from breast cancer patients who underwent Taxol-based neoadjuvant chemotherapy (NAC) with response (Grade 5, sensitive to Taxol-based chemotherapy) or without response (Grade 2, insensitive to Taxol-based chemotherapy), followed by immunohistochemistry staining for cleaved caspase 3, EGR1 and Bax (a well-known pro-apoptotic protein related to EGR1). The results showed that the expression levels of EGR1 and cleaved caspase 3 were significantly higher in the Taxol chemo-sensitive Grade 5 than that in the Taxol chemo-insensitive Grade 2 (Figure 7E, supplementary Figure S9). These results support the dependency of Taxol efficacy on caspase-mediated Asp^132^-cleavage of AURKA for apoptosis. Thus, AURKA cleavage provides a potential key biomarker for the efficacy of clinical cancer chemotherapy.

## Discussion

Cancer is one of the leading causes of death worldwide. In addition to surgery and radiotherapy, chemotherapy is the first line of treatment for many cancers 45. Understanding the mechanism of chemotherapeutic drugs may enhance the efficacy of anti-cancer therapies and help overcome drug resistance. We demonstrate for the first time that AURKA, a key serine/threonine kinase in mitosis, undergoes proteolytic cleavage at Asp^132^ in various cancer cells in response to apoptotic stimuli. The cleavage of AURKA at Asp^132^ is induced by caspase 3, 7, and 8 during cell apoptosis. Both the N-terminal and C-terminal cleaved products of AURKA at Asp^132^ elicit cell apoptosis by inducing centrosome and spindle assembly defects. The D132A mutation of AURKA blocks the activation of cleaved caspase 3 and EGR1 expression, reducing the sensitivity of cancer cells to the chemotherapeutic drug Taxol *in vitro* and *in vivo*. The sequence around the Asp^132^-cleavage site within AURKA is highly conserved in different mammalian species, highlighting the significance of this cleavage site. It is also worthy to mention that bigger fragment size of exogenous AURKA^D132^ cleavage in rat KMM cells than that in human BCBL cells, indicating that AURKA may undergo post-translational modification in rat cells, and shed a light on that D132 cleavage is highly conserved in different species no matter the modification of AURKA occur or not.

In addition to apoptotic stimuli, AURKA is cleaved in response to viral infection. The KSHV-encoded antigen LANA not only induces Asp^76^-cleavage of AURKB, leading to cell progression 34, but also induces AURKA cleavage at Asp^132^ to activate cell apoptosis. Thus, the viral oncoprotein may play a critical role in dynamically controlling the fate of host cells during the life cycle of viral infection by selectively inducing cleavage of AUKRA or AUKRB in response to intracellular and extracellular stress, and indicating that AURKA and AURKB undergo proteolytic cleavage at similar sites (Asp^132^ and Asp^76^) is fully precisely regulated at the post-translational level to mediate viral-mediated oncogenesis. In addition to targeting key host proteins for cleavage, the oncoprotein LANA undergoes caspase cleavage to suppress apoptosis and inflammation 46. Overall, these findings indicate that proteolytic cleavage is fully hijacked by viruses.

Colony formation was significantly decreased in KMM and HeLa cells expressing AURKA^1-132^ and AURKA^133-end^ compared with colony formation in cells expressing AURKA^WT^. This supports the notion that AURKA proteolysis at Asp^132^ enhances cell death. Moreover, the AURKA^D132^ mutation attenuates the sensitivity of cells to Taxol compared to AURKA^WT^* in vitro and in vivo*, suggesting that the Taxol exerts anti-cancer effects by inducing Asp^132^-cleavage of AURKA. Our findings suggest that mutation of AURKA^D132^ or caspase 3/7/8 reduces the efficacy of chemotherapeutic drugs and may serve as predictive biomarkers for drug resistance during cancer patient chemotherapy.

Taxol is a well-known drug approved by the US Food and Drug Administration (FDA) as an anti-mitotic chemotherapy for the treatment of a variety of cancers, including ovarian cancer, AIDS-related Kaposi's sarcoma, cervical cancer, and breast cancer 1. However, the emergence of drug resistance during chemotherapy is a major challenge that limits the effectiveness and clinical application of Taxol. For example, only about 50% of breast cancer patients achieve tumor regression in response to Taxol treatment 47. The discovery of caspase-mediated Asp^132^-cleavage of AURKA provides an alternative strategy to overcome resistance and improve the efficacy of chemotherapeutic drugs that contain Taxol. The positive relationship between the apoptotic markers (cleaved caspase 3 and EGR1) and the efficacy of Taxol chemotherapy in breast cancer patients, highlights the role of Asp^132^-cleavage of AURKA.

Paclitaxel exerts cytotoxicity by binding to the β-subunit of tubulin and preventing tubulin depolymerization to inhibit mitosis 48. In addition, paclitaxel exerts antitumor effects via multipolar spindle and chromosomal instability 49; however, the molecular mechanisms for this process are unclear. We demonstrate that Asp^132^-cleavage of AURKA in response to Taxol induces cell apoptosis, providing a clear molecular mechanism for the effects of Taxol. Several potential mechanisms for Taxol resistance have been identified, including overexpression of ABC transporters that promote drug efflux, and the expression of different β-tubulin subtypes 50, however, the relationship between these mechanisms and Asp^132^-cleavage of AURKA is unclear.

Apoptosis, a form of programmed cell death, plays a central role in eliminating damaged, infected, and unwanted cells from the body. Apoptosis can be divided into an intrinsic pathway (the mitochondrial pathway) and an extrinsic pathway (the death receptor pathway) 2. Tight regulation of apoptosis is crucial for normal development and homeostasis in multicellular organisms. However, dysregulation of apoptosis, particularly the activation of anti-apoptotic systems, can result in uncontrolled cell proliferation and contribute to tumor progression. Caspases are central to the control of cell apoptosis, and can be classified into three groups according to difference of their structure and function, including inflammatory caspases (caspase 1, 4, 5, and 11), apoptotic initiator caspases (caspase 2, 8, 9, and 10), and apoptotic executioner caspases (caspase 3, 6, and 7) 51. Both initiator and executioner caspases are initially synthesized as inactive zymogens, which are activated under specific conditions. In response to apoptotic cell death signals, initiator caspases undergo auto-activation through auto-proteolysis, resulting in the activation of downstream or executioner caspases to execute apoptotic functions 52. Caspase 3 deficiency in MCF-7 breast cancer cells attenuates apoptosis in response to chemotherapeutic drugs, suggesting that lack of caspase 3 expression contributes to chemo-resistance 53. Our findings reveal that the Asp^132^-cleavage product of AURKA induces cell apoptosis in a caspase 3/7/8-dependent manner, highlighting the cooperation of caspases in regulating cell fate.

AURKA is a key kinase in centrosome maturation and separation during cell division. Centrosome consists of pairs of centrioles, which are surrounded by electron-dense pericentriolar material and function as a major microtubule-organizing center to ensure accurate chromosome segregation 54. Therefore, centrosome abnormalities, including amplification and structural defects, cause chromosomal instability 55. We demonstrate that both Asp^132^-cleaved products (1-132, and 133-end) of AURKA cause centrosome and spindle assembly defects, in agreement with the previous discovery that Taxol exerts its cytotoxicity by inducing multipolar spindles 49.

In summary, the Asp^132^-cleavage of AURKA can be induced in various human cancer cells by Taxol in a caspase 3/7/8-dependent manner, and the Asp^132^-cleavage products cause centrosome and spindle assembly defects during mitosis to induce cell apoptosis. Mutation of the AURKA cleavage site suppresses Taxol-induced apoptosis *in vitro* and *in vivo*, providing a potential biomarker for chemotherapeutic drug efficacy.

## Supplementary Material

Supplementary figures and tables.
