# Supplementary material for: Caspase-mediated AURKA cleavage at Asp132 is essential for paclitaxel to elicit cell apoptosis
Source: Theranostics. 2024 Jun 17;14(10):3909–26. doi: 10.7150/thno.97842 (PMC11234276; doi:10.7150/thno.97842)
Supplement: Supplementary file 1 — Supplementary figures and tables. [file thnov14p3909s1.pdf]

Supplemental Figures and Tables

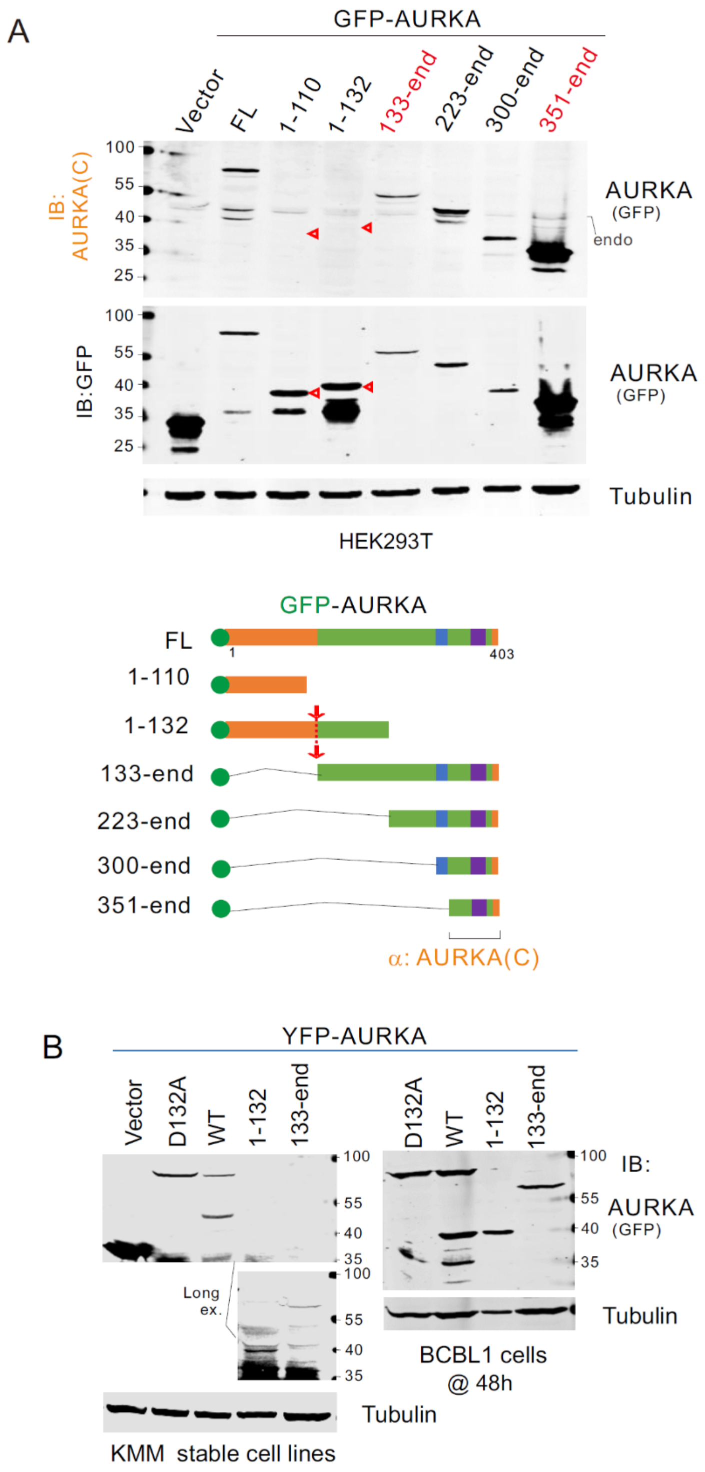

Figure S1. (A) The epitope of AURKA recognized by antibodies against AURKA(C). Related to Figure 1. HEK293T cells were transfected with GFP-tagged full-length (FL) AURKA or the truncated mutants as indicated in the figure. At 48 h post-transfection, cell lysates were subjected to

immunoblotting (IB) with the indicated antibodies. Bottom panel, a schematic of wild-type AURKA and the truncated mutants with GFP tags at the N-terminus.

(**B**) **Establishment of KSHV-infected cell lines stably expressing YFP-tagged AURKA. Related to Figure 1.** Equal numbers of KMM stably expressing YFP-tagged vector alone, wild-type (WT) AURKA, or the D132A, N-cleaved (1-132), or C-cleaved (133-end) mutants, or BCBL1 cells transiently expression at 48 h post-transfection, were subjected to immunoblotting analysis with the indicated antibodies (GFP-tag antibodies were used to detect exogenous AURKA).

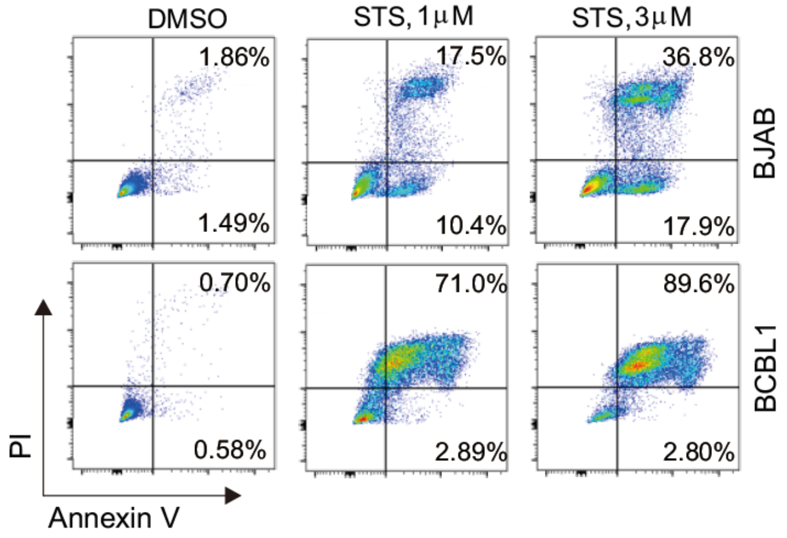

Figure S2. The sensitivity of KSHV-infected BCBL1 and uninfected BJAB cells in response to STS-induced apoptosis. Equal numbers of cells were treated with STS (0, 1, and 3 μM) for 3 h, followed by staining with Annexin-V-FITC and propidium iodide (PI) for flow cytometry analysis. **Related to Figure 2.**

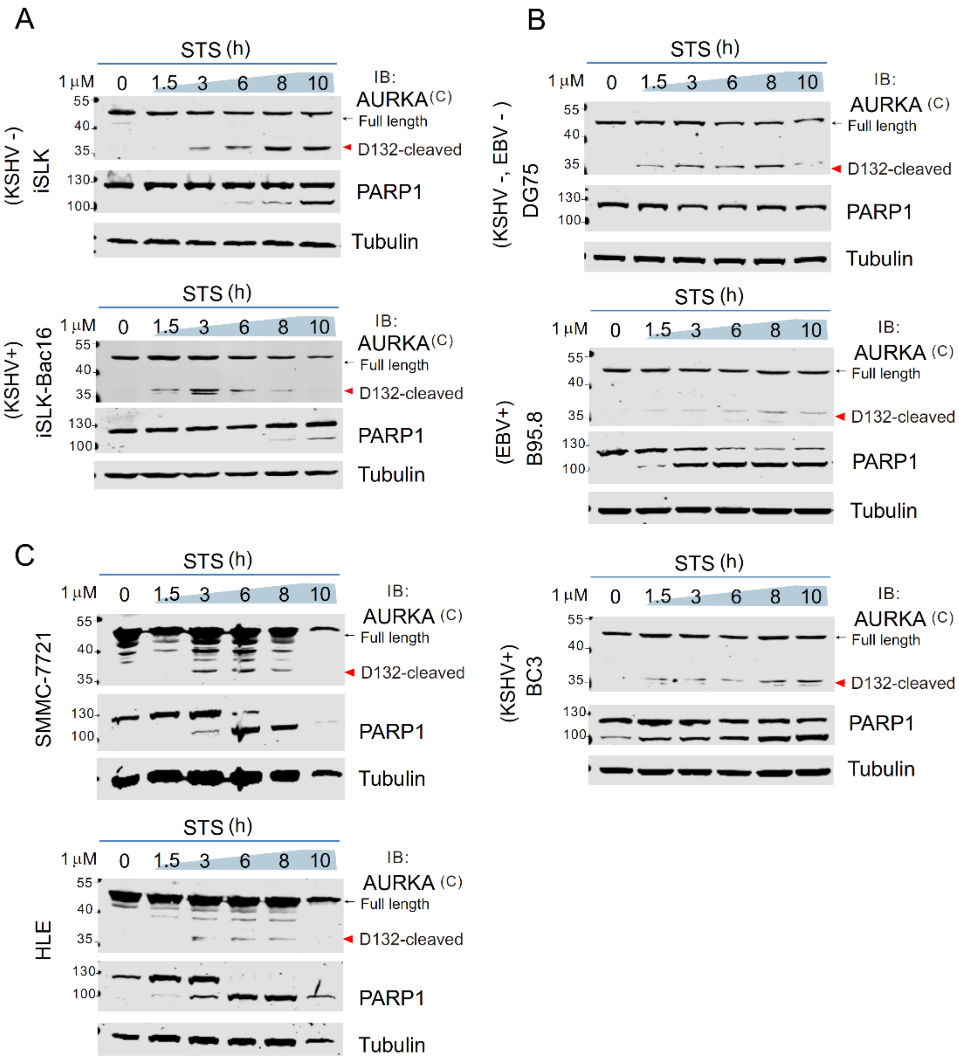

Figure S3. Asp^{132} cleavage of AURKA occurs in different cancer cell lines with or without viral infection in the presence of STS-induced apoptosis. Equal numbers of (**A**) KSHV-infected iSLK-Bac16 and uninfected iSLK epithelial cells, (**B**) EBV-infected B95.8, KSHV-infected BC3, and EBV/KSHV uninfected DG75 B-lymphoma cells, and (**C**) liver cancer cells (SMMC-7721 and HLE), were individually stimulated with STS (1 μM) for the indicated time, followed by immunoblotting analysis with the indicated antibodies. Tubulin was used as an internal control. The arrow indicates the Asp^{132}-cleavage product of AURKA. **Related to Figure 2.**

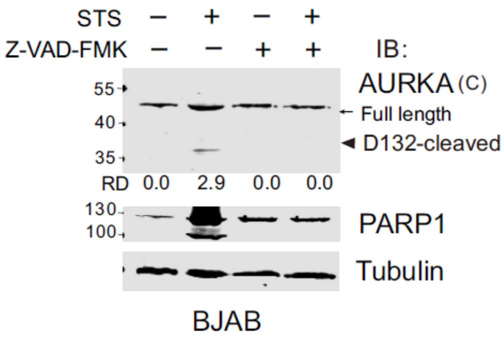

Figure S4. The Asp[132] cleavage of AURKA induced by STS is blocked by the pan-caspase inhibitor (Z-VAD-FMK). Equal numbers of BJAB cells were preincubated with Z-VAD-FMK (25 μM) for 1 h, and then treated with STS (1 μM) for 6 h, followed by immunoblotting analysis with antibodies as indicated. **Related to Figure 3**.

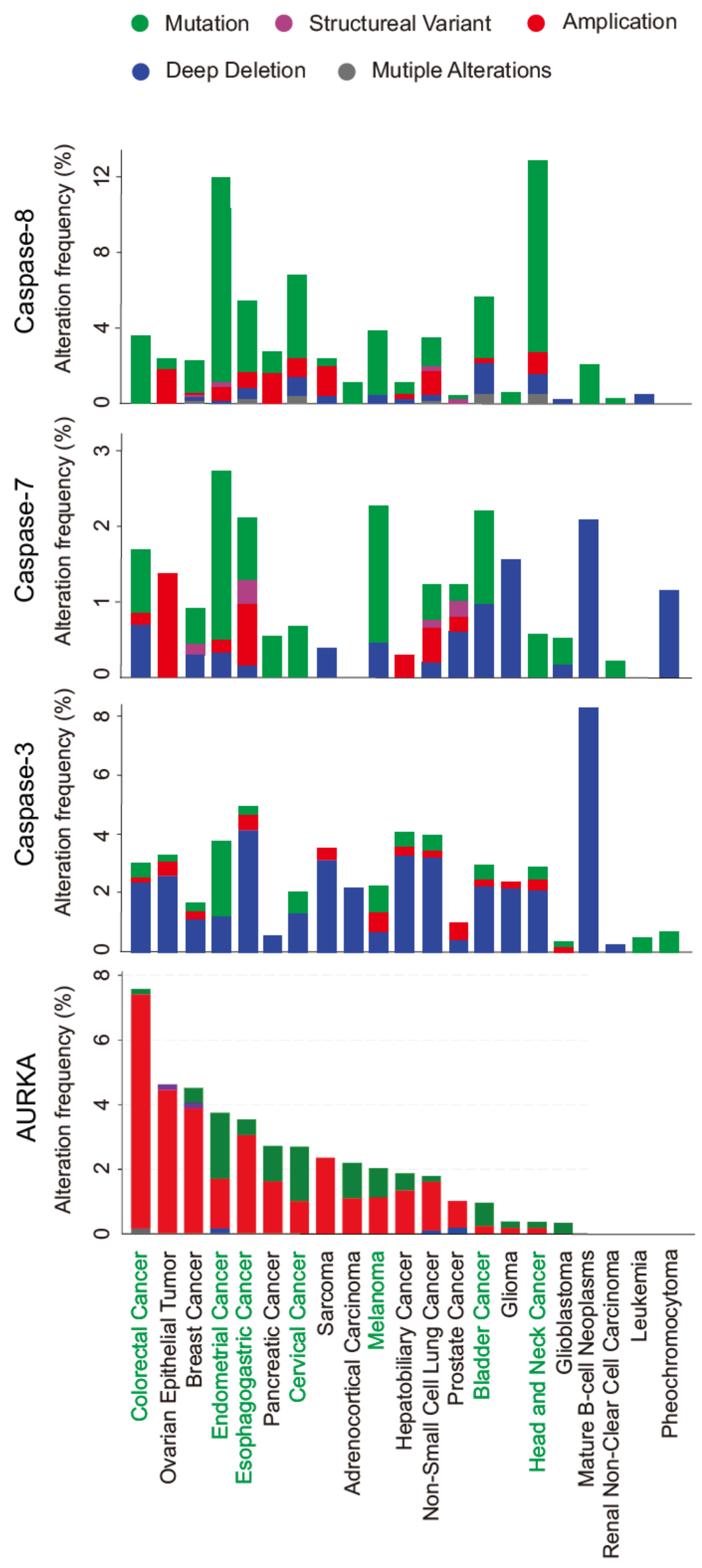

Figure S5. The amplification frequency of AURKA correlates with caspase 3, 7, or 8 mutations in most cancers. The alteration frequency of AURKA and caspase 3, 7, and 8 was analyzed using the cBioPortal database online. **Related to Figure 3**.

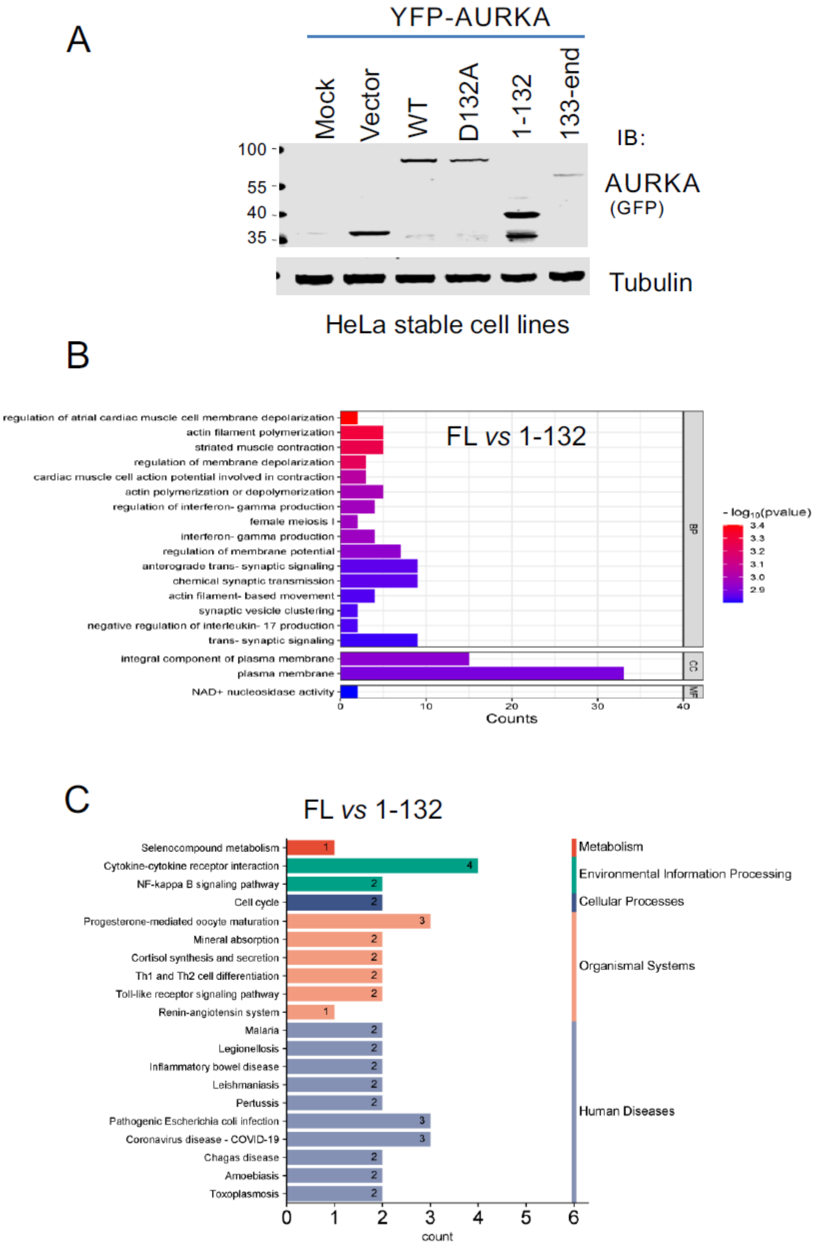

Figure S6. (**A**) Generation of HeLa cell lines stably expressing YFP-tagged AURKA. Equal numbers of HeLa parental (Mock) cells and cells stably expressing the vector only, YFP-tagged wild-type (WT) AURKA or the D132A, N-cleaved (1-132), and C-cleaved (133-end) mutants, were subjected to immunoblotting analysis with the indicated antibodies. **Related to Figure 4.** (**B**) GO functional enrichment analysis of differentially expressed genes (DEGs) between AURKA^{FL} and AURKA$^{1\text{-}132}$ from *panel B* of Figure 4. **Related to Figure 4.** (**C**) KEGG enrichment analysis of DEGs from AURKA^{FL} vs. AURKA$^{1\text{-}132}$ from *panel B* of Figure 4. **Related to Figure 4.**

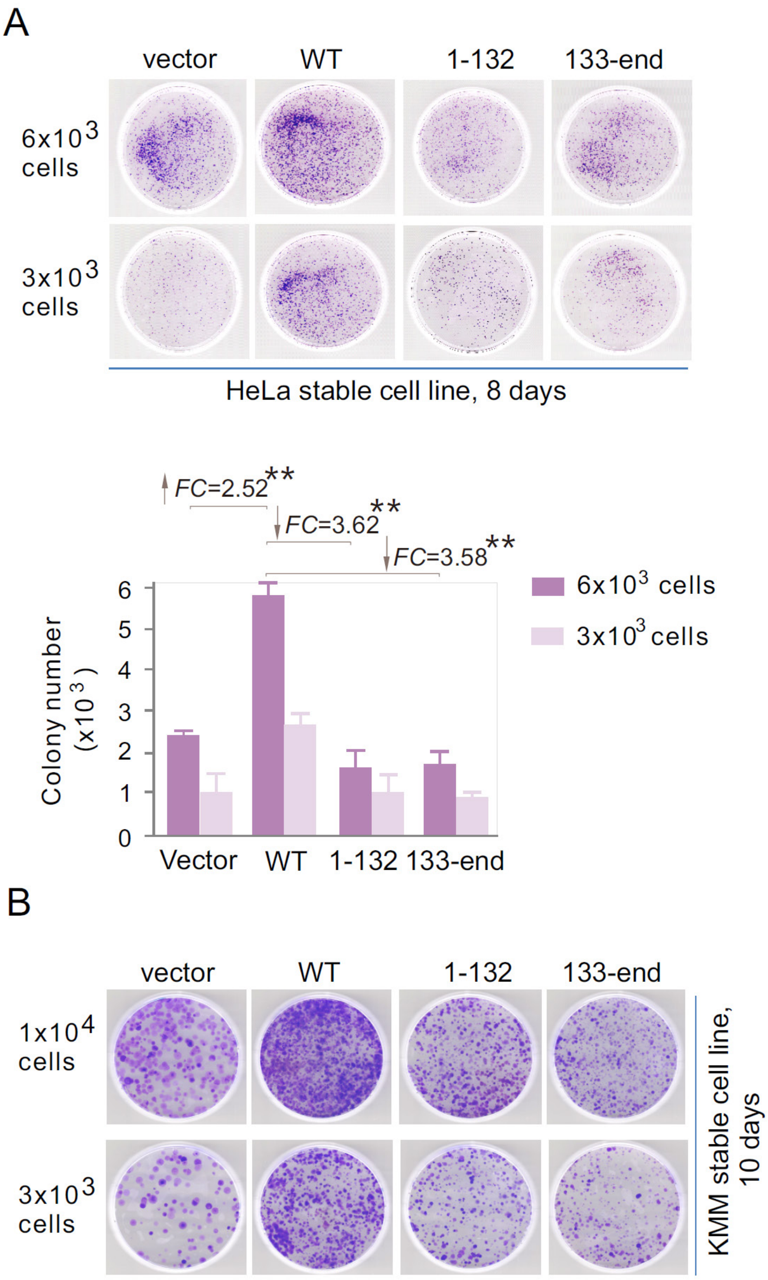

Figure S7. Asp[132]-cleaved product of AURKA reduces colony formation *in vitro*. Equal numbers of (**A**) HeLa or (**B**) KMM cells stably expressing wild-type (WT) AURKA or the N-terminus (1–132) and C-terminus (133–end) mutants were treated with 1 μg/mL puromycin, and then fixed 8 or 10 days later followed by staining with crystal violet to determine the colony numbers. The relative colony formation levels were calculated from three independent experiments. **, p<0.01. FC, fold change. **Related to Figure 7.**

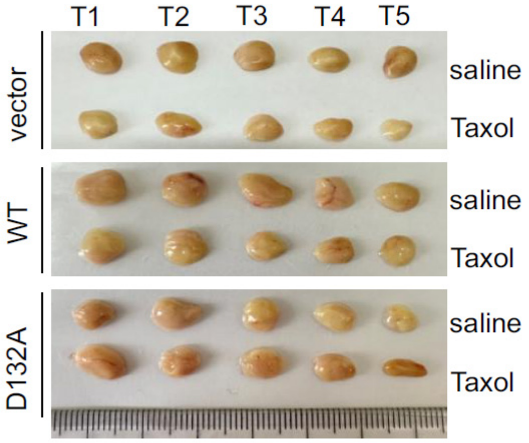

Figure S8. Taxol reduces the growth of tumor cells expressing wild-type AURKA but not the D132A mutant *in vivo*. Photographs of tumor tissues induced by HeLa cells stably expressing the vector only, wild-type AURKA, or the D132A mutant in nude mice from Figure 7B. **Related to Figure 7.**

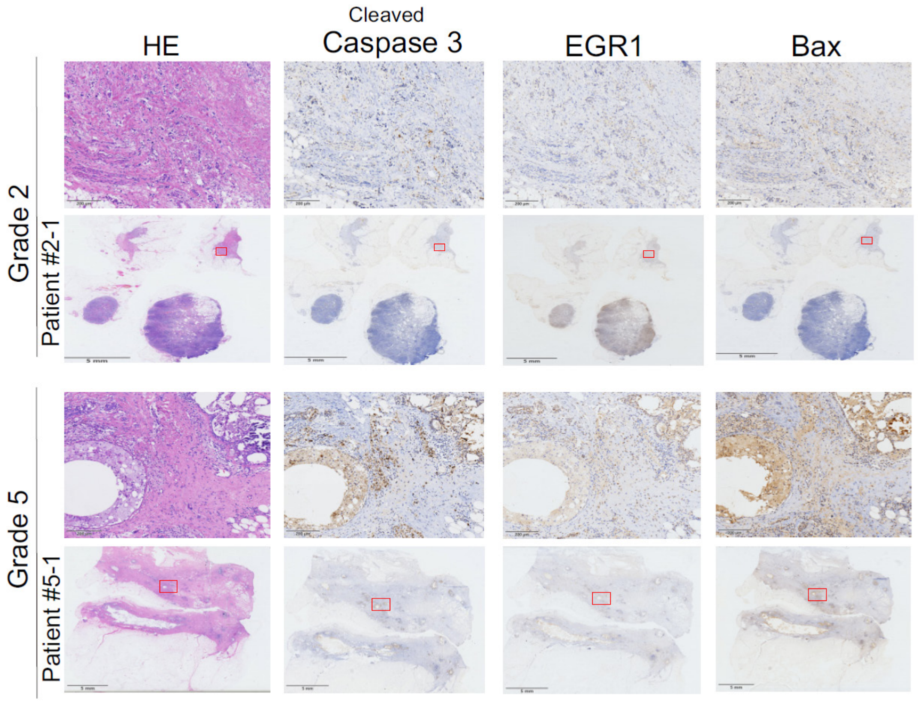

Figure S9. Representative images of patient tissues of grade 2 or 5 group immunostained with antibodies for cleaved caspase 3 or EGR1. Enlarged views are shown in the top panels. **Related to Figure 7.**

Table S1 Sample Information from breast cancer patients. Related to Figure 7.

Group	Patient (record No.)	Age		Therapeutic Strategy
Grade 2	Patient #2-1 (2323045)	66	TP	T（Abraxane）400mg iv drip d1<br>P（Carboplatin）550mg iv drip d1
Grade 2	Patient #2-2 (2322314)	73	THP	T（Abraxane）400mg iv drip d1<br>H (Trastuzumab) 560mg iv drip d1<br>P (Pertuzumab) 840mg iv drip d1
Grade 2	Patient #2-3 (2322109)	31	TEC	T（Abraxane）400mg iv drip d1<br>E (Pirarubicin) 130mg iv drip d1<br>C (Cyclophosphamide) 850mg iv drip d1
Grade 2	Patient #2-4 (2322061)	60	TAC	T（Abraxane）100mg iv drip d1<br>A (Epirubicin) 90mg iv drip d1<br>C (Cyclophosphamide) 700mg iv drip d1
Grade 2	Patient #2-5 (2321223)	60	TEC	T（Abraxane）400mg iv drip d1<br>E (Doxorubicin) 40mg iv drip d1<br>C (Cyclophosphamide) 800mg iv drip d1
Grade 2	Patient #2-6 (2320723)	60	T	T（Abraxane）400mg iv drip d1
Grade 2	Patient #2-7 (2311943)	49	TEC	T（Abraxane）400mg iv drip d1<br>E (Epirubicin) 120mg iv drip d1<br>C (Cyclophosphamide) 850mg iv drip d1
Grade 2	Patient #2-8 (2310240)	46	TAC	T (Paclitaxel) 330mg iv drip d1<br>A (epirubicin) 120mg iv drip d1<br>C (Cyclophosphamide) 800mg iv drip d1
Grade 2	Patient #2-9 (2309310)	43	TCbHP	T (Abraxane) 400mg iv drip d1<br>Cb (carboplatin) 750mg iv drip d1<br>H (Trastuzumab) 400mg iv drip d1<br>P (Pertuzumab) 420mg iv drip d1
Grade 5	Patient #5-1 (2323103)	45	TCbHP	T（Abraxane） 400mg iv drip d1<br>Cb (carboplatin) 650mg iv drip d1<br>H (Trastuzumab) 432mg iv drip d1<br>P (Pertuzumab) 840mg iv drip d1
Grade 5	Patient #5-2 (2322801)	48	TEC	T (Abraxane) 130mg iv drip d1<br>E (Epirubicin) 130mg iv drip d1<br>Cb (carboplatin) 700mg iv drip d1
Grade 5	Patient #5-3 (2320411)	47	TCbHP	T (Abraxane) 120mg iv drip d1<br>Cb (carboplatin) 450mg iv drip d1<br>H (Trastuzumab) 320mg iv drip d1<br>P (Pertuzumab) 420mg iv drip d1
Grade 5	Patient #5-4 (2319977)	33	TEC	T (Abraxane) 400mg iv drip d1<br>E (Epirubicin) 120mg iv drip d1<br>C (Cyclophosphamide) 800mg iv drip d1
Grade 5	Patient #5-5	41	TCbHP	T (Abraxane) 400mg iv drip d1

	(2319631)			Cb (carboplatin) 750mg iv drip d1<br>H (Trastuzumab) 480mg iv drip d1<br>P (Pertuzumab) 840mg iv drip d1
Grade 5	Patient #5-6<br>(2319585)	50	TEC	T (Abraxane) 400mg iv drip d1<br>E(Liposome doxorubicin) 40mg iv drip d1<br>C (Cyclophosphamide) 900mg iv drip d1
Grade 5	Patient #5-7<br>(2319096)	38	THP	T（Abraxane) 360mg iv drip d1<br>H (Trastuzumab) 380mg iv drip d1<br>P (Pertuzumab) 840mg iv drip d1
Grade 5	Patient #5-8<br>(2319072)	30	TCbHP	T（Abraxane) 400mg iv drip d1<br>Cb (carboplatin) 800mg iv drip d1<br>H (Trastuzumab) 480mg iv drip d1<br>P (Pertuzumab) 840mg iv drip d1
Grade 5	Patient #5-9<br>(2317203)	41	TEC	T（Abraxane) 380mg iv drip d1<br>E (Liposome doxorubicin) 40mg iv drip d1<br>C (Cyclophosphamide) 700mgiv drip d1

Table S2 Oligonucleotides. Related to Figure 3 and 4.

Oligonucleotides	Source	Identifier
Forward primer for Caspase 3-sgRNA-1:<br>5’-CACCGTACCCGGGTTAACCGAAAGG-3’	This paper	Custom synthesis
Reverse primer for Caspase 3-sgRNA-1:<br>5’-AAACCCTTTCGGTTAACCCGGGTAC-3’	This paper	Custom synthesis
Forward primer for Caspase 3-sgRNA-2：<br>5‘-CACCGACTGGAATGACATCTCGGTC-3’	This paper	Custom synthesis
Reverse primer for Caspase 3-sgRNA-2:<br>5‘-AAACGACCGAGATGTCATTCCAGTC-3’	This paper	Custom synthesis
Forward primer for Caspase 6-sgRNA-1:<br>5’-CACCGGAAAGTTTCTCAGCGCCGAG-3’	This paper	Custom synthesis
Reverse primer for Caspase 6-sgRNA-1:<br>5‘-AAACCTCGGCGCTGAGAAACTTTCC-3’	This paper	Custom synthesis
Forward primer for Caspase 6-sgRNA-2:<br>5‘-CACCGATAGAGACAATCTTACCCGC-3’	This paper	Custom synthesis
Reverse primer for Caspase 6-sgRNA-2:<br>5‘-AAACGCGGGTAAGATTGTCTCTATC-3’	This paper	Custom synthesis
Forward primer for Caspase 7-sgRNA-1：<br>5‘-CACCGATGGCATCCAGGCCGACTCG-3’	This paper	Custom synthesis
Reverse primer for Caspase 7-sgRNA-1：<br>5‘-AAACCGAGTCGGCCTGGATGCCATC-3’	This paper	Custom synthesis
Forward primer for Caspase 7-sgRNA-2：<br>5‘-CACCGGGGACGGTACAAACGAGGAC-3’	This paper	Custom synthesis
Reverse primer for Caspase 7-sgRNA-2：	This paper	Custom synthesis

5‘-AAACGTCCTCGTTTGTACCGTCCCC-3’		
Forward primer for Caspase 8-sgRNA-1：<br>5‘-CACCG GTGAGAATATCATCGCCTCG-3’	This paper	Custom synthesis
Reverse primer for Caspase 8-sgRNA-1：<br>5‘-AAAC CGAGGCGATGATATTCTCACC-3’	This paper	Custom synthesis
Forward primer for Caspase 8-sgRNA-2：<br>5‘-CACCGGCCTGGACTACATTCCGCAA-3’	This paper	Custom synthesis
Reverse primer for Caspase 8-sgRNA-2：<br>5‘-AAACTTGCGGAATGTAGTCCAGGCC-3’	This paper	Custom synthesis
Forward primer for β-actin qPCR:<br>5‘-GCCGCCAGCTCACCAT-3’	This paper	Custom synthesis
Reverse primer for β-actin qPCR:<br>5’-TCGTCGCCCACATAGGAATC-3’	This paper	Custom synthesis
Forward primer for FOS qPCR:<br>5’-TGGCGTTGTGAAGACCATGA-3’	This paper	Custom synthesis
Reverse primer for FOS qPCR:<br>5’-AGTTGGTCTGTCTCCGCTTG-3’	This paper	Custom synthesis
Forward primer for FOSB qPCR:<br>5’-ACCGACTCCAGGCGGAGA-3’	This paper	Custom synthesis
Reverse primer for FOSB qPCR:<br>5’-GAGAGAAGCCGTCAGGTTGG-3’	This paper	Custom synthesis
Forward primer for Jun qPCR:<br>5’-CAGCCAGGTCGGCAGTATAG-3’	This paper	Custom synthesis
Reverse primer for Jun qPCR:<br>5’-GGACTCTGCCACTTGTCTCC-3’	This paper	Custom synthesis

Forward primer for EGR1 qPCR: 5’-CACCTGACCGCAGAGTCTTT-3’	This paper	Custom synthesis
Reverse primer for EGR1 qPCR: 5’-CTGACCAAGCTGAAGAGGGG-3’	This paper	Custom synthesis
Forward primer for CCN1 qPCR: 5’-AGCAGCCTGAAAAAGGGCAA-3’	This paper	Custom synthesis
Reverse primer for CCN1 qPCR: 5’-AGCCTGTAGAAGGGAAACGC-3’	This paper	Custom synthesis
Forward primer for CCN2 qPCR: 5’-ATGGTGCTCCCTGCATCTTC-3’	This paper	Custom synthesis
Reverse primer for CCN2 qPCR: 5’-TCTTCCAGTCGGTAAGCCGC-3’	This paper	Custom synthesis
